# An Extended Entropic Model for Cohesive Sediment Flocculation in a Piecewise Varied Shear Environment

**DOI:** 10.3390/e23101263

**Published:** 2021-09-28

**Authors:** Zhongfan Zhu, Jie Dou

**Affiliations:** 1 Beijing Key Laboratory of Urban Hydrological Cycle and Sponge City Technology, College of Water Sciences, Beijing Normal University, Beijing 100875, China; 2 Three Gorges Research Center for Geo-Hazards, Ministry of Education, China University of Geosciences, Wuhan 430074, China; douj888@gmail.com

**Keywords:** cohesive sediment, flocculation, entropy, piecewise varied shear, model

## Abstract

In this study, an extended model for describing the temporal evolution of a characteristic floc size of cohesive sediment particles when the flocculation system is subject to a piecewise varied turbulent shear rate was derived by the probability methods based on the Shannon entropy theory following Zhu (2018). This model only contained three important parameters: initial and steady-state values of floc size, and a parameter characterizing the maximum capacity for floc size increase (or decay), and it can be adopted to capture well a monotonic pattern in which floc size increases (or decays) with flocculation time. Comparison with 13 literature experimental data sets regarding floc size variation to a varied shear rate showed the validity of the entropic model with a high correlation coefficient and few errors. Furthermore, for the case of tapered shear flocculation, it was found that there was a power decay of the capacity parameter with the shear rate, which is similar to the dependence of the steady-state floc size on the shear rate. The entropic model was further parameterized by introducing these two empirical relations into it, and the finally obtained model was found to be more sensitive to two empirical coefficients that have been incorporated into the capacity parameter than those in the steady-state floc size. The proposed entropic model could have the potential, as an addition to existing flocculation models, to be coupled into present mature hydrodynamic models to model the cohesive sediment transport in estuarine and coastal regions.

## 1. Introduction

Cohesive sediment is composed of water, fine-grained sediments (such as silt and clay), and organic matter [1,2,3,4], and it can absorb some pollutants (such as heavy metals) and nutrients on its surface due to a short-range electrochemical attraction [5,6,7]. When some cohesive sediments are transported into rivers, lakes, reservoirs, and estuarine and coastal waters, they are aggregated due to a particle–particle attractive force, forming some flocs of varied sizes; however, they also experience a breakage impact in the turbulent flow when the flow shear exceeds their inherent strengths [8,9,10]. Flocs are greatly different from primary particles (i.e., the basic element of which a porous floc is comprised) in terms of larger sizes, more porous structures, and faster-settling velocities [4,11,12,13]. Therefore, studying the flocculation characteristic of cohesive sediment particles is of importance since it plays an essential role in affecting the water quality change, ecosystem function evolution, and bio-geochemical cycle process in some aquatic environments [4,14,15,16,17].

Flocculation of cohesive particles is not only limited to cohesive sediment science. In other research areas, such as sanitary engineering, water treatment works, and colloidal science, particle flocculation is an essential element in some dynamic processes, and related studies regarding its mechanism are always a research focus [18,19,20,21,22,23,24,25,26,27,28,29]. Especially, the temporal evolution of the floc size distribution of cohesive particles in a turbulent fluid has been investigated by many researchers via theoretical formulation [2,9,30,31,32], numerical simulation [33,34,35,36,37,38,39], and experimental observation [6,23,26,40] in many disciplines. At present, there are three kinds of deterministic flocculation models in the literature, as summarized by Shen et al. (2015) [37]. The first kind is the Lagrangian flocculation model originally proposed by Winterwerp (1998) [9] and its modified versions [2,28,31,32]. The second kind of flocculation model is the population balance model (PBM) [10,34,36,37,38,41], which is based on a classic equation originally by Smoluchowski (1918) [34,42,43,44]. The third kind of flocculation model is based on the lattice Boltzmann method [37,39,45].

In contrast to the above deterministic models, there has also been a kind of flocculation model in a stochastic form based on some probability knowledge [46,47,48]. By considering a sequence of stochastic aggregation and breakup events among particles, Maggi (2008) formulated a stochastic Lagrangian model to describe the flocculation of suspended cohesive sediment flocs in water [46]. This model can be adopted to reflect floc mobility within the population size spectrum in terms of a stochastic perspective. Different from Son and Hsu (2009) [30], Shin et al. (2015) dealt with the floc breakup term of the Winterwerp model to be a stochastic variable, and the modified model can reproduce the floc size spectrum well for different water–sediment conditions [47]. Zhu (2018) derived a simple and explicit expression for floc size variation in a fixed flow shear environment based on the entropy concept. Recently, Shen et al. (2021) developed a quasi-Monte Carlo model to predict the temporal evolution of floc size distribution of cohesive sediment in aquatic environments, and its validity has been tested by comparing with known simple analytical solutions and two series of laboratory experimental data [48].

These models have provided a tool to predict the floc size distribution (or characteristic floc size) in a constant turbulent shear environment. However, different from a laboratory experiment where a simple flow shear condition has been easily controlled [28,49,50,51], hydrodynamic conditions in natural waters are always complicated [17,52,53,54,55,56]. For example, it could include many cycles of a high and low shear condition, which leads to a complicated variation of floc size distribution. Some in situ observations have shown that floc size distribution is sensitive to the change of flow shear rate in a tidal estuary, deltas, and coastal regions [52,53,55,57]. Eisma and Li (2019) and Braithwaite et al. (2012) observed an obvious difference in floc size distribution among the slack, ebb, and flood parts of a tidal cycle in the estuary [28,58,59]. In river mouths, some measurements have also shown that river input condition reshapes the floc size distribution in waters [28,55,60]. In the wastewater treatment, sanitary engineering, and colloidal science fields, the flocs are often subject to cycles of high and low shearing conditions to maximize the flocculation efficiency and optimize the separation effect [22,24]. Thus, for these engineering circumstances, attention should be paid to the performance of flocculation models for cohesive particle flocculation under a piecewise varied shearing condition in terms of accuracy and efficiency.

In this study, we attempted to extend the work of Zhu (2018) and proposed a simple flocculation model of cohesive particles in a consecutive varied shear environment using the entropic model. Different from existing flocculation models, the entropic model contains a small number of input parameters and calibrated parameters, and it might be an attractive choice for tracing the floc size variation in a complicated hydrodynamic condition. Entropy, as a measure of uncertainty associated with the system, originates from the thermodynamic subject and is widely applied to various research fields [61]. In hydraulic engineering, Chiu (1987) was the first to derive the one-dimensional longitudinal velocity distribution along the vertical direction in an open channel using the entropy concept [62]. Since then, some researchers have adopted the entropy method based on the probability to deal with some classic hydraulic engineering problems. They include velocity distribution in open channels or a canopy open channel flow [63,64], suspended sediment concentration distribution [65], boundary shear stress in circular and trapezoidal channels [66], infiltration process in unsaturated soils [67], flow duration curve [68], sediment graph [69], rating curve [70], and velocity-dip position in an open channel [71]. In these works, the entropic method showed its potential to tackle the engineering problems as an addition to existing deterministic models.

The paper is arranged as follows. Starting from a single-step flocculation model, Section 2 formulates a multi-step model for flocculation of cohesive particles subject to a piecewise varied shear using the Shannon entropy concept. Section 3 tests the model accuracy by comparing it with 13 experimental data sets regarding floc size variation in a varied shear condition from the literature. Two important parameters that have been incorporated into the model, the steady-state floc size and the maximum capacity for floc size growth (or decay) and the sensitivity of the entropic model to some empirical parameters, as well as an application of the entropic model in engineering practices, are also analyzed in Section 4. Finally, Section 5 presents some concluding remarks.

## 2. Multi-Step Entropic Model for Sediment Flocculation

The turbulent flow environment where sediment flocculation develops is often characterized by the flow shear rate G (its unit is s^−1^). It is defined as G=$\sqrt{\varepsilon /\nu }$, where ε is the turbulent dissipation rate of flow and ν is the kinematic viscosity of the fluid, as employed by some studies [9,10,21,23,26,27,28,40,72].

Since the flocculation model for a constant shear environment is the basis for the flocculation model formulation for a piecewise varied shear condition, a constant shear-induced flocculation model using the entropy method is presented firstly in the following.

### 2.1. Flocculation Model for a Constant Flow Shear Environment

#### 2.1.1. Floc Size Growth toward the Steady State at a Constant Shear Rate

**Figure 1:**
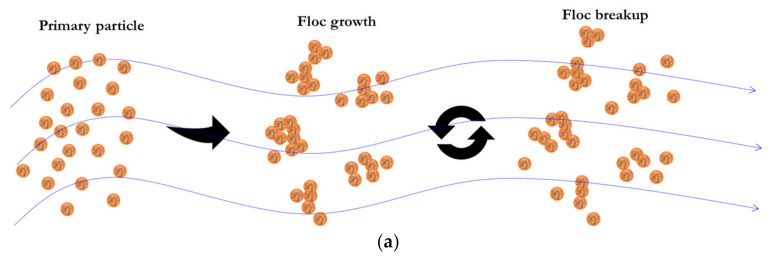

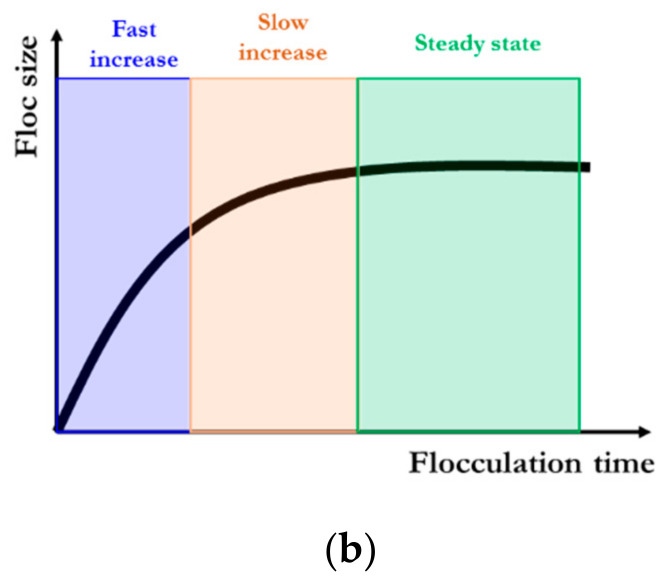
Schematic of particle flocculation in a constant shear rate (partly redrawn based on Zhu (2018) [73]): (**a**) floc variation; (**b**) floc size variation with flocculation time.

As shown in Figure 1, the sediment flocculation system experiences a rapidly increasing phase, a subsequently slowly increasing phase, and finally a steady state in terms of the characteristic size of the floc population (always characterized by its median size of floc population, L) with flocculation time t, given a constant flow shear environment (i.e., constant G). The application of the Shannon entropic method to the flocculation process includes the following steps: definition of Shannon entropic function, specification of constraint condition, maximization of entropic function, calculation of Lagrange multiplier, hypothesis on a cumulative distribution function, and finally derivation of flocculation model, as presented in Zhu (2018) [73]. To avoid repetition, the results for flocculation expression are only presented here in this study, and specific mathematical derivations regarding it can be found in Zhu (2018) [73] in more detail.

By the Shannon entropy method, floc size $L\left(t\right)$ can be obtained as:

(1) $$L\left(t\right)=L_{s}-\left(L_{s}-L_{0}\right)\exp\left[-\frac{L_{s}-L_{0}}{M}\left(t-t_{0}\right)\right]$$

where $L_{0}$ and $L_{s}$ are the initial value and the steady-state value of floc size, respectively, M is the proposed maximum capacity for floc size growth (its unit is equal to the multiplication of units of floc size and flocculation time), and $t_{0}$ is the initial time at which flocculation starts. It can be seen from Equation (1) that floc size growth approaches its steady state almost after a flocculation time point $t_{0}+3M/\left(L_{s}-L_{0}\right)$ for a given flocculation system, and, in other words, the proposed parameter M determines the rapidity with which the steady state is approached. In the work of Zhu (2018) [73], Equation (1) was presented to agree with existing experimental data sets well, and the parameter M is a monotonic function of the flow shear rate G, which will be mentioned in Section 4.1.

#### 2.1.2. Floc Size Decay toward Another Steady State at a Higher Constant Shear Rate

**Figure 2:**
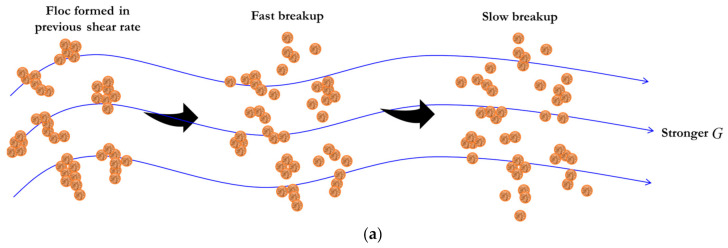

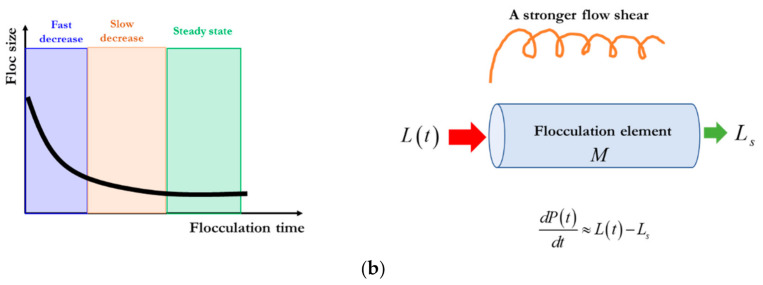
Schematic of floc size decay subject to a stronger flow shear: (**a**) floc variation; (**b**) floc size variation with flocculation time and flocculation element.

When the flocculation system is suddenly subject to a stronger shear, floc size will make a corresponding response. As flocculation time progresses, floc size experiences a rapidly decreasing phase and a slowly decreasing phase before entering another steady state, as shown by some laboratory experiments [18,19,20,22,23,24,27,74]. As illustrated in Figure 2, we can also derive an entropic flocculation model for this case, which is similar to that mentioned in Section 2.1.1.

The Shannon entropy function H(L) of floc size during stronger shear-induced flocculation can be written in the following form [62]:

(2) $$H(L)=-\int_{L_{s}}^{L_{0}}f(L)[\ln f(L)]dL$$

where f(L) is the probability density function, and the probability law becomes:

(3) $$\int_{L_{s}}^{L_{0}}f(L)dL=1$$

where $L_{s}$< $L_{0}$. Furthermore, the Lagrangian function Λ is constructed by

(4) $$\Lambda =-\int_{L_{s}}^{L_{0}}f(L)[\ln f(L)]dL+\lambda \left[\int_{L_{s}}^{L_{0}}f(L)dL-1\right]$$

where λ is the Lagrange multiplier. Taking its derivative to f(L) and defining it to be zero can still have:

(5) $$f(L)=\exp\left(\lambda -1\right)$$

Integrating it from the steady-state value $L_{s}$ to arbitrary value L can yield $F\left(L\right)$ in the following form:

(6) $$F(L)=\left(L-L_{s}\right)\exp\left(\lambda -1\right)$$

Choose a small flocculation element at a certain flocculation time t (as shown in Figure 2b) with its input being floc size $L\left(t\right)$ and its output being approximated by its steady-state value $L_{s}$. Here, the parameters P and M denote the cumulative quantity and the maximum capacity for floc size decay, respectively. The mass conservation equation for the flocculation element becomes

(7) $$\frac{dP\left(t\right)}{dt}\approx L\left(t\right)-L_{s}$$

Further, the cumulative distribution function of floc size in the space domain could be hypothesized to be 1 minus the ratio of the cumulative quantity of floc size decay to the maximum capacity, i.e.,

(8) $$F\left(L\right)=1-\frac{P}{M}$$

which means the cumulative distribution function monotonically increases from null to unity as flocculation time progresses from the initial time to infinity, and all of the values of flocculation time t between $t_{0}$ and ∞ are equally likely.

Substituting Equation (5) into Equation (3) can have: $\exp\left(\lambda -1\right)=1/\left(L_{0}-L_{s}\right)$. Combining it with Equations (6)–(8) can yield the following analytical expression for floc size $L\left(t\right)$ after using the initial condition ($L=L_{0}$ at $t=t_{0}$):

(9) $$L\left(t\right)=L_{s}+\left(L_{0}-L_{s}\right)\exp\left[-\frac{L_{0}-L_{s}}{M}\left(t-t_{0}\right)\right]$$

It can be seen that Equations (1) and (9) are a little different in terms of the numerator term in the exponential function. Thus, they can be generalized to be:

(10) $$L\left(t\right)=L_{s}-\left(L_{s}-L_{0}\right)\exp\left[-\frac{\left|L_{s}-L_{0}\right|}{M}\left(t-t_{0}\right)\right]$$

### 2.2. Flocculation Model for a Piecewise Varied Shear

**Figure 3:**
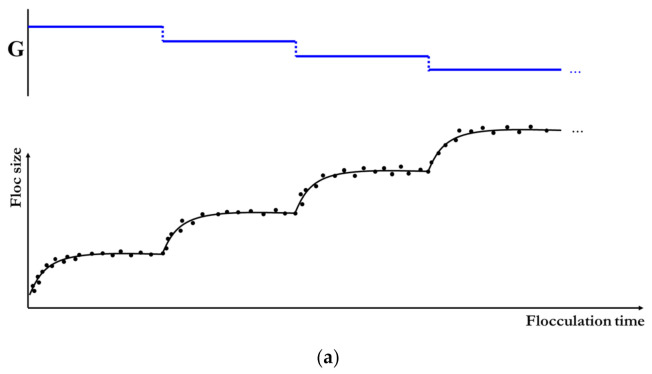

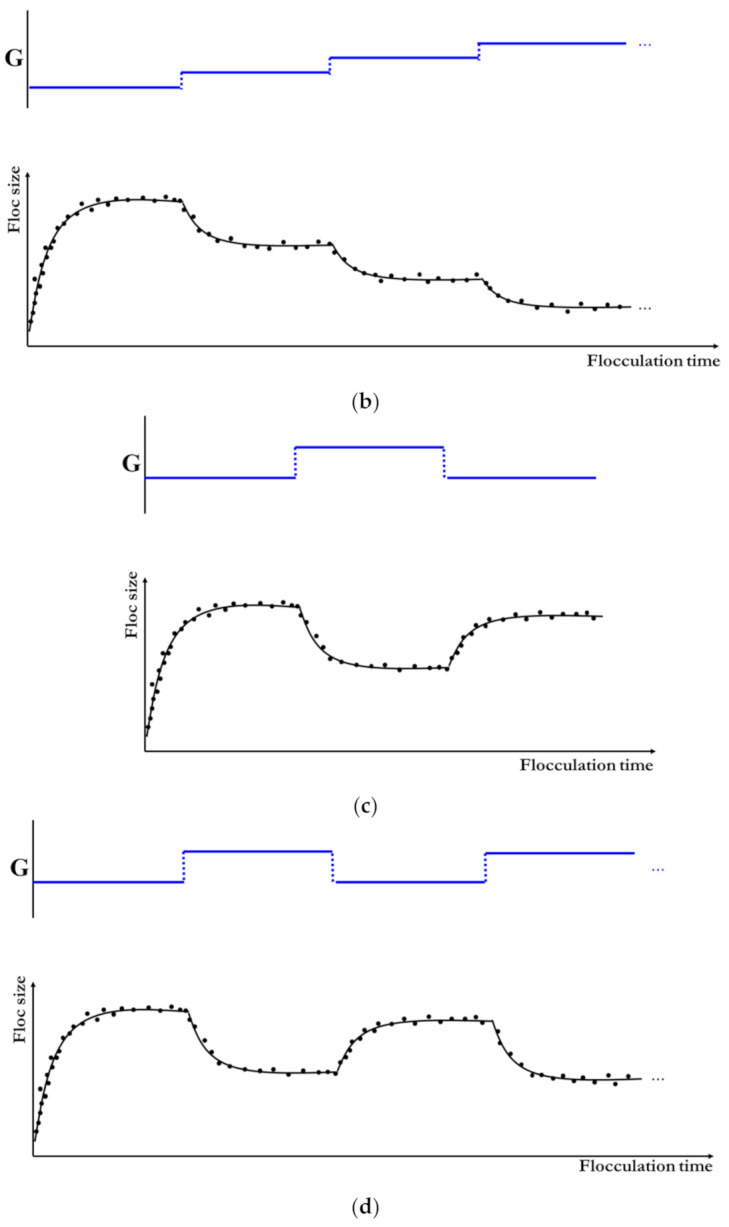
Floc size variation subject to a piecewise varied shear rate: (**a**) a piecewise increasing shear; (**b**) a piecewise decreasing shear; (**c**) sequenced flow shear (low shear → high shear → low shear); (**d**) consecutive cycled flow shear (low shear → high shear → low shear → high shear …).

When the flocculation system is subject to a piecewise varied shear, floc size is inferred to show a piecewise exponential increase (or decrease) behavior with flocculation time [18,20,22,23,27,28]. Figure 3a–d schematically shows four different types of floc size variation to various types of piecewise varied shear environments, which is similar to a sediment accumulation effect in reservoirs, dams, and river channels [75,76]. They include a piecewise increasing flow shear, a piecewise decreasing flow shear, sequenced flow shear (e.g., low shear → high shear → low shear), and consecutive cycled flow shear (e.g., low shear → high shear → low shear→ high shear…).

For each type of piecewise varied shear, Equation (10) still holds for each stage where the flow shear rate is a constant. Consider a n*-step* shear procedure: $G=G_{i}$ at $t_{i-1}\leq t<t_{i}$, i=1, 2, … n, where $G_{1},G_{2},\ldots ,G_{n}$ are piecewise values of the shear rate, and $t_{0},t_{1},\ldots ,t_{n}$ are piecewise time nodes. For the i*-th* flocculation period, the temporal evolution of floc size $L_{i}\left(t\right)$ can be described by Equation (10). However, $L_{s}$ in Equation (10) should be substituted by $L_{s,i}$, the steady-state value of floc size in the i-step flocculation period, $L_{1}$ by $L_{i-1}$, a floc size value at $t=t_{i-1}$, and M by $M_{i}$ corresponding to a constant $G_{i}$ period, respectively. Therefore, Equation (10) can be recast in the following form:

(11) $$L_{i}\left(t\right)=L_{s,i}-\left(L_{s,i}-L_{i-1}\right)\exp\left[-\frac{\left|L_{s,i}-L_{i-1}\right|}{M_{i}}\left(t-t_{i-1}\right)\right]$$

For example, for three piecewise shear schedules, $G=G_{1},G_{2},G_{3}$ at $t_{0}\leq t\leq t_{1}$, $t_{1}\leq t\leq t_{2}$, and $t_{2}\leq t\leq t_{3}$, respectively, Equation (11) can be expanded into the following recursive formula for floc size variation:

(12) $$\left\{\begin{array}{c} L_{1}\left(t\right)=L_{s,1}-\left(L_{s,1}-L_{0}\right)\exp\left[-\frac{\left|L_{s,1}-L_{0}\right|}{M_{1}}\left(t-t_{0}\right)\right]\\ L_{2}\left(t\right)=L_{s,2}-\left[L_{s,2}-L_{1}\left(t_{1}\right)\right]\exp\left[-\frac{\left|L_{s,2}-L_{1}\left(t_{1}\right)\right|}{M_{2}}\left(t-t_{1}\right)\right]\\ L_{3}\left(t\right)=L_{s,3}-\left[L_{s,3}-L_{2}\left(t_{2}\right)\right]\exp\left[-\frac{\left|L_{s,3}-L_{2}\left(t_{2}\right)\right|}{M_{3}}\left(t-t_{2}\right)\right] \end{array}\right.$$

which can be adopted to predict the temporal evolution of floc size in a three piecewise shear environment provided the values of $L_{s,1}$, $L_{s,2}$, $L_{s,3}$ and $M_{1}$, $M_{2}$, $M_{3}$ are known prior.

## 3. Test with Experimental Data

### 3.1. Performance Evaluation of the Model

To evaluate the accuracy of the proposed entropic model with experimental data points, an error analysis was performed in this study, including the following three statistical parameters:

(1) Correlation coefficient *R*^2^ between the observed data points and the modeled data points;
(2) The average relative error (*RE*) between the observed data points and the modeled data, which is calculated as follows: $\frac{1}{N}\sum_{i=1}^{N}\frac{\left|y_{Oi}-y_{Mi}\right|}{y_{Oi}}$, where $y_{Oi}$ and $y_{Mi}$ are the observed data and the modeled data, and N is the total number of data points;
(3) The root mean square error (*RMSE*) between the observed data points and the modeled data points: *RMSE*=$\sqrt{\frac{1}{N}\sum_{i=1}^{N}{\left(y_{Oi}-y_{Mi}\right)}^{2}}$.

### 3.2. Comparison with Experimental Data Sets in the Literature

Experimental data sets regarding floc size variation in a varied flow shear environment are collected as much as possible in the literature, covering different flocculation materials and various flocculation environments in many subjects. They include three experimental data sets in the cohesive sediment field, Burban et al. (1989) [50], Keyvani and Strom (2014) [28], and Tsai et al. (1987) [77]; eight experimental data sets in the water treatment field, Biggs et al. (2003) [18], Chaignon et al. (2002) [27], Gregory (2004) [19], Nan et al. (2016) [20], Slavik et al. (2012) [22], Wu et al. (2019) [29], Xu and Gao (2012) [74], and Xu et al. (2010) [24]; and two experimental data sets in the colloidal science field, Spicer et al. (1998) [23] and Wu and van de Ven (2009) [25].

#### 3.2.1. Cohesive Sediment Field

The effect of flow shear on the flocculation dynamic of fine-grained sediments in freshwater was studied in the experiment of Tsai et al. (1987) [77]. The sediments were collected from the Detroit River inlet of Lake Erie, and the Couette viscometer was adopted to generate the turbulence environment. Shear stresses were set to be 1, 2, and 4 dynes/cm^2^, corresponding to shear rates of 100, 200, and 400 s^−1^, and sediment concentrations ranged from 50, 100, 400, and 800 mg/L. In this experiment, floc size distribution as a function of flocculation time was recorded. Experimental observations showed that the steady-state floc size decreased with shear stress and that they increased with sediment concentration.

Using the same flocculation apparatus as those in Tsai et al. (1987) [77], Burban et al. (1989) [50] investigated experimentally and theoretically the effects of salinity, fluid shear rate, and sediment concentration on the flocculation of fine-grained sediment from the Detroit River. The adopted shear rates were from 100 to 600 s^−1^, whereas the sediment concentration covered from 10 to 800 mg/L. Experimental data regarding floc size distributions as a function of flocculation time were obtained. Based on experimental results, physical mechanisms regarding the impact of salinity, fluid shear, and sediment concentration on the aggregation and breakup of cohesive sediment were analyzed in their work.

A laboratory study of Keyvani and Strom (2014) [28] focused on the effects of repeated exposure to multiple cycles of high and low shearing conditions on the growth pattern and the steady-state size of mud flocs. The flocculation material was a 50 mg/L mixture of kaolinite and montmorillonite clay, whereas a 13-L mixing chamber (27.5 cm × 27.5 cm × 25 cm) with a rotating paddle as well as a high-resolution imaging system formed an environment for the flocculation observation. The flocs were allowed to grow until an equilibrium was reached at a shear rate of 35 s^−1^, and they were broken down with a strong turbulent shear of 400 s^−1^. This procedure was repeated seven times in their experiment, and the characteristics of the floc population (floc size distribution, floc circularity index, and floc number variation) were measured and analyzed. Their observations demonstrated that initial particle size distributions play little role in the steady-state floc size, whereas it plays an important role in the flow growth rate. The flocs become also slightly stronger and less reactive with repeated cycles of growth and breakup.

**Figure 4:**
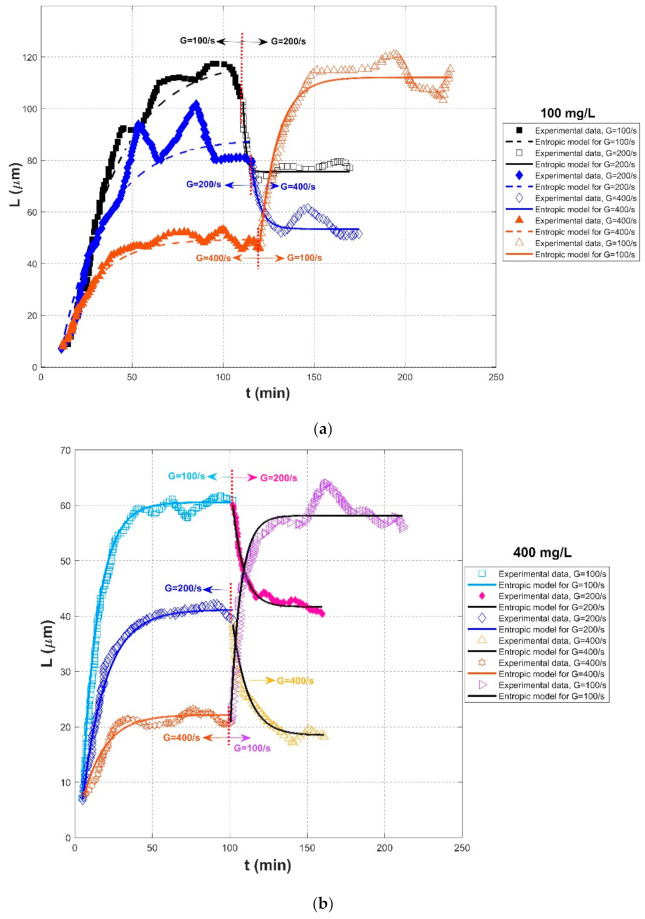
Comparison of the entropic model with experimental data points of Tsai et al. (1987) [77]: (**a**) 100 mg/L floc concentration; (**b**) 400 mg/L floc concentration.

**Figure 5:**
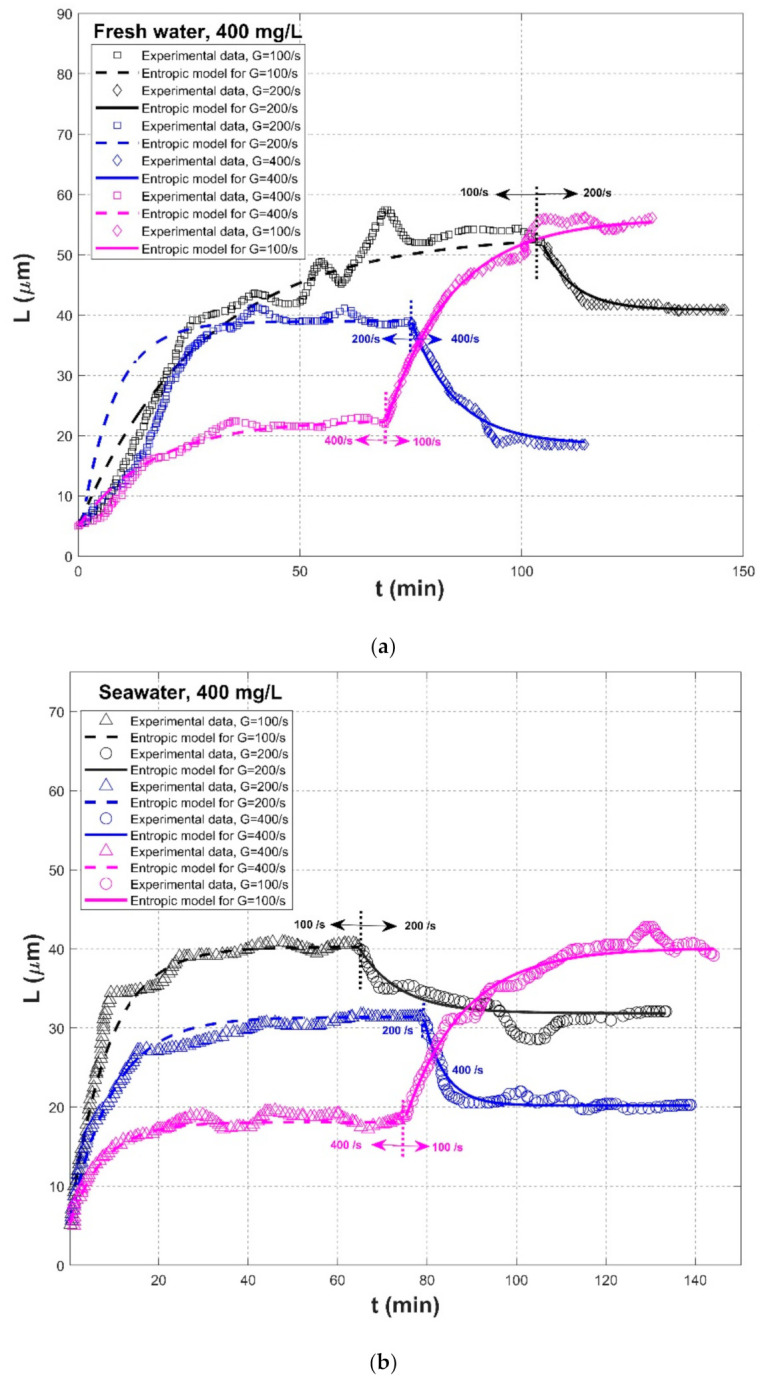
Comparison of the entropic model with experimental data points of Burban et al. (1989) [50]: (**a**) fresh water at a concentration of 400 mg/L; (**b**) seawater at a concentration of 400 mg/L.

**Figure 6:**
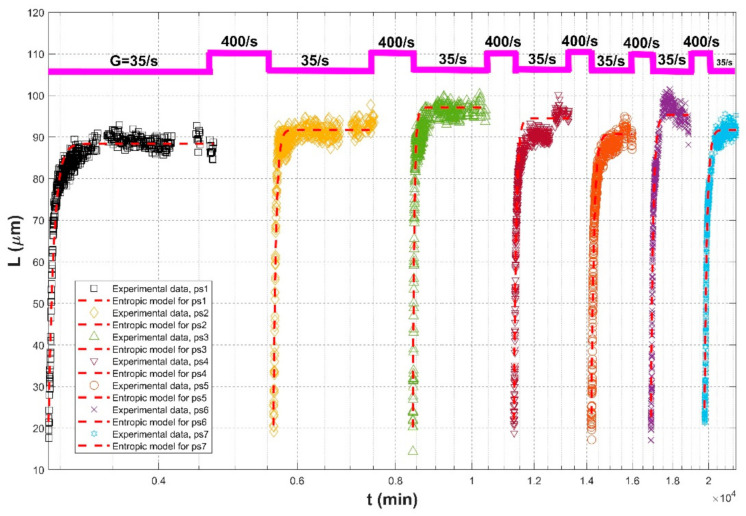
Comparison of the entropic model with experimental data points of Keyvani and Strom (2014) [28]. Here ps 1 refers to prior shear 1, etc., ps 7 refers to prior shear 7.

Figure 4, Figure 5 and Figure 6 show comparisons of the proposed entropic model (Equation (11)) with experimental data of Tsai et al. (1987) [77], Burban et al. (1989) [50], and Keyvani and Strom (2014) [28], respectively. Table 1 presents these comparison results. In this table, the second through fifth columns present the experimental conditions adopted in these experiments, including flocculation material, turbulence-generated apparatus, flocculation conditions, and the adopted shear rates, respectively. The values of $L_{0}$ and $L_{s}$ in the sixth and seventh columns were obtained from experimental data. Fitting effects of the entropic model for these experimental results, including the calculated values of *R*^2^, *RE*, *RMSE*, are listed in the last three columns, as well as the values M in the eighth column.

**Figure 7:**
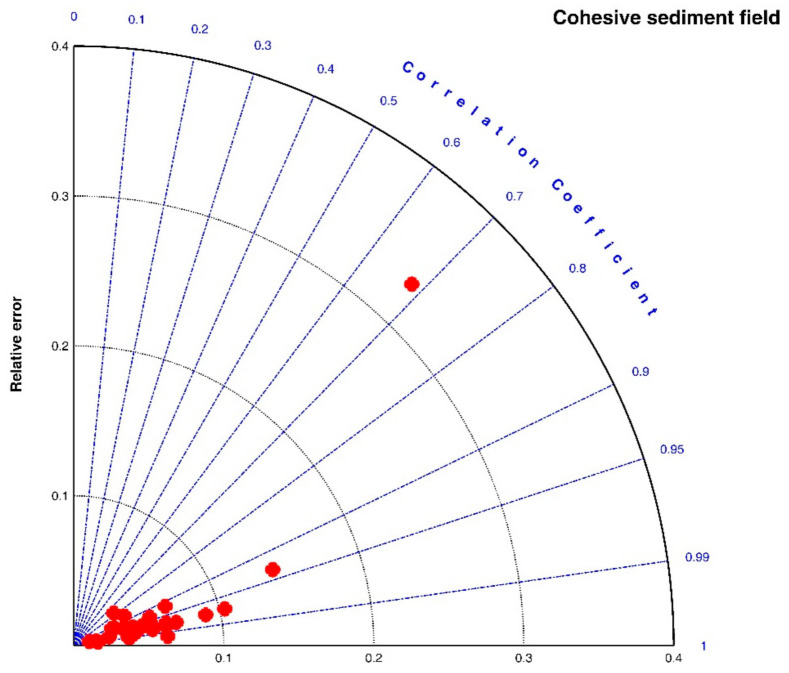
Taylor diagram of calculated correlation coefficients *R*^2^ and relative errors *RE* for experimental cases in the cohesive sediment field.

Figure 7 shows the Taylor diagram of calculated correlation coefficients and relative errors for all of the cases. It can be seen that the entropic model can fit most experimental data sets with a high correlation coefficient above 0.9 and a relative error below 0.1. As presented in the last row of Table 1, the average values of *R*^2^, *RE*, and *RMSE* for all experimental data are 0.9453, 0.0595, 2.8560, respectively. The largest deviation in Figure 7 comes from the comparison of the entropic model with experiment data points of G = 200 s^−1^ in the case of freshwater flocculation in Burban et al. (1989), as shown by the blue color in Figure 5a. The reason is that the floc size experienced a slow growth process before reaching the steady state, for which the model did not track it well. Additionally, a large relative error can be seen from Figure 5a for G = 200 s^−1^ in the case of 100 mg/L sediment concentration in Tsai et al. (1987) and from Figure 6a for G = 100 s^−1^ in the case of fresh water flocculation in Burban et al. (1989) since there was a strong data scatter that may have been due to measurement uncertainties. Additionally, the entropic model seemed to work better with seawater sediment flocculation by comparing Figure 5a,b, which might be attributed to a smaller data scatter for seawater sediment flocculation relative to freshwater sediment.

As shown by Figure 4a,b and Figure 5a,b, an abrupt change of the shear rate triggered the flocculation system that had already reached an equilibrium to evolve toward another equilibrium, for which the entropic model can be also applicable. In Figure 6, due to a lack of experimental data regarding floc breakage at a high shear rate G of 400 s^−1^, the entropic model could only fit those flow growth data; although some data were crowding in each period, an agreement between the proposed model and original experimental points was still noticeable.

**Table 1:** Summary of the comparison result of the entropic model with experimental data sets regarding floc size variation subject to a varied shear rate collected in the cohesive sediment field. The last row shows the average values of statistical errors.

References	Flocculation Experiment Condition						Fitting Effect			
	Material	Flocculation Environment	Flocculation Condition	Flocculation Period	*L*_0_ (μm)	*L_s_* (μm)	*M* (μm*min)	*R* ^2^	*RE*	*RMSE*
Tsai et al. (1987) [77]	Sediment from the Detroit River	Couette Viscometer	100 mg/L Sediment concentration	*G* = 100/s	8.89	118.75	3000	0.9754	0.0928	5.8169
				*G* = 200/s	102.75	75.62;	40	0.9177	0.0295	3.0310
				*G* = 200/s	7.06	88.43	2000	0.9341	0.1419	8.6894
				*G* = 400/s	78.69	53.40	120	0.8586	0.0390	3.0530
				*G* = 400/s	7.84	49.48	780	0.9796	0.0537	2.2237
				*G* = 100/s	46.27	112.11	650	0.9544	0.0413	4.5201
			400 mg/L Sediment concentration	*G* = 100/s	7.11	60.54	560	0.9953	0.0629	2.1620
				*G* = 200/s	60.01	41.67	130	0.9799	0.0152	0.8524
				*G* = 200/s	6.83	41.15	520	0.9890	0.0380	1.2036
				*G* = 400/s	38.45	18.47	200	0.9606	0.0476	1.7866
				*G* = 400/s	7.69	22.19	210	0.9182	0.0663	1.2766
				*G* = 100/s	20.85	58.14	240	0.9607	0.0473	2.6358
Burban et al. (1989) [50]	Natural bottom sediments from the Detroit River	Horizontal Couette type flocculator	Sediments in fresh water at a concentration of 400 mg/L.	*G* = 100/s	5	53.37	1350	0.9715	0.1036	3.2498
				*G* = 200/s	51.52	40.85	75	0.9665	0.0106	0.6365
				*G* = 200/s	5.46	38.95	250	0.6827	0.3300	8.2543
				*G* = 400/s	38.68	18.56	200	0.9815	0.0394	1.0488
				*G* = 400/s	5	22.60	300	0.9735	0.0903	1.3645
				*G* = 100/s	22.44	56	500	0.9894	0.0163	1.0980
			Sediments in seawater at a concentration of 400 mg/L.	*G* = 100/s	5	40.25	300	0.9768	0.0428	1.7719
				*G* = 200/s	40.00	31.86	75	0.7741	0.0343	1.4053
				*G* = 200/s	5	31.39	250	0.9880	0.0367	1.0293
				*G* = 400/s	30.83	20.19	50	0.9111	0.0276	0.8020
				*G* = 400/s	5	18.09	100	0.9781	0.0380	0.6666
				*G* = 100/s	18.89	40.12	280	0.9698	0.0242	1.1663
Keyvani and Strom (2014) [28]	80% kaolinite and 20% montmorillonite	Mixing chamber with a rotating paddle	G = 35/s for floc growth followed by G = 400/s for 15 h breakup, repeated 7 times.	*G* = 35/s, ps1	21.45	88.38	2800	0.9459	0.0349	3.1614
				*G* = 35/s, ps2	20.60	91.74	3000	0.9401	0.0361	3.4063
				*G* = 35/s, ps3	20.20	97.07	3000	0.9361	0.0540	4.9519
				*G* = 35/s, ps4	21.92	94.47	5500	0.9708	0.0633	4.8604
				*G* = 35/s, ps5	23.40	90.69	7500	0.9728	0.0545	3.6739
				*G* = 35/s, ps6	22.58	95.35	8500	0.9752	0.0699	4.6352
				*G* = 35/s, ps7	24.76	91.73	9500	0.9784	0.0626	4.1021
**In average**								**0.9453**	**0.0595**	**2.8560**

#### 3.2.2. Water Treatment Field

Using local, activated sludge samples from a wastewater treatment plant in Maxeville, France, the flocculation experiment of Chaignon et al. (2002) [27] focused on the effect of cycled-shear conditions on the evolution of average floc size. Sludge flocculation occurred in a 1-L baffled reactor (90 mm × 150 mm) with a 15 mm × 54 mm blade, and an adjustable-speed motor was set to provide a large shear rate range from 135 to 370 s^−1^. Two experimental conditions including raw sludge of 35 mg/L and sludge spiked with 20 wt% of aquatic particles were monitored and compared in their study.

In Biggs et al. (2003) [18], activated sludge collected from a wastewater treatment plant in Australia was adopted to carry out the flocculation experiment in a 1.2-L baffled batch vessel with a flat six-blade impeller. Malvern Mastersizer/E instrument was used to measure the real-time floc size variation. Two groups of experimental procedures were arranged to investigate the effect of shear history on floc size. They included a procedure (E1), a shear process with G = 19.4 s^−1^ for 85 min (floc aggregation), G = 113 s^−1^ for 35 min (floc breakup), and G = 19.4 s^−1^ again for floc re-growth; and another cycling shear procedure (E2), two consecutive step changes in the shear rate G between 19.4 and 113 s^−1^.

Flocculation of kaolin suspension (50 mg/L) from London tap water in a stirred jar was investigated in Gregory (2004) [19]. Three kinds of coagulants were added into the suspension, including alum and two commercial poly aluminum chloride (PACl) products, XL-1: r’ = 1.9, 5.1 wt.% Al and XL-9: r’ = 2.1, 4.6 wt.%, where r’ (=OH/Al) denotes the different degrees of neutralization. In this study, the flocculation index (dimensionless) of the suspension instead of floc size distribution, which is an empirical index of floc size, was monitored by an optical measurement system. The adopted shear rate procedures were (1) G = 11 s^−1^ for 600 s, followed by G = 340 s^−1^ for 60 s and again G = 11 s^−1^, and (2) G = 23 s^−1^ followed by a stronger G = 520 s^−1^ and a returning G = 23 s^−1^. Their experimental results showed that PACl yields gave larger flocs than with alum. For the alum coagulant, floc breakage was not fully reversible; however, for the case of polyDADMAC (a high-charge, low-molecular-weight cationic polyelectrolyte) added into the suspension, floc breakage after experiencing a strong shear condition reduced to the original one before a high shear rate to a large degree.

Xu et al. (2010) [24] performed a series of flocculation experiments to study the effects of varied shear rates and solution pH on aggregation and breakage of humic acid flocs in 1-L cylindrical plexiglass beakers using a conventional Jar-test apparatus. Two kinds of coagulants, poly aluminum chloride (PAC) and the Al_13_O_4_ (OH)_24_^7+^ (Al_13_) polymer, were added into the suspension, and the floc size distribution was monitored timely using a laser diffraction instrument (Malvern Mastersizer 2000). Since some authors have asserted that a global shear rate in the jar does not represent the flow shear condition for determining floc size distribution, the stirring speed r was directly adopted to characterize the shear condition in their study. The stirring speed ranged from 40 rpm for floc growth to 200 rpm for floc breakage, and there were two groups of shear procedures: (1) r = 40 rpm for floc growth → different stirring rates for breakup → r = 40 rpm for floc regrowth, and (2) r = 40 rpm for floc growth → r = 200 rpm for breakup with pH = 5, 7, 9 → r = 40 rpm for floc regrowth. Experimental results demonstrated that the flocs formed by Al_13_ polymer were weaker than those of PAC, whereas Al_13_ polymer showed a better recoverability than PAC. Additionally, the pH of the solution had an impact on floc size and floc recoverability. Furthermore, Xu and Gao (2012) [74] focused on the three kinds of Al-based coagulants, including alum, PACl, and PACl–Al_b_, on the floc formation, breakage, and re-growth profiles of humic acid flocs. In this study, three shear rates were adopted for floc breakage: G = 34.6, 87.8, and 223.5 s^−1^, and G = 10.1 s^−1^ was fixed for floc formation and floc re-growth. The impact of floc breakage for 5 min and 10 min at a fixed G = 87.8 s^−1^ on the floc size profile was also compared in their experiment.

Effects of shear stress and increases in pH on the evolution of the Fe-dissolved organic matter (DOM) and Al-DOM flocs in raw water were identified in the experimental study of Slavik et al. (2012) [22]. The flocculation test was performed in 2-L beakers with baffles, and floc size was monitored using an improved light transmission technique with dynamic extinction measurement. Four shear rates were adopted: G = 40, 500, 1000, and 1500 s^−1^. Three experimental cases were included: (1) G = 40 s^−1^ for floc growth → G = 500, 1000, and 1500 s^−1^ for floc breakage → G = 40 s^−1^ again for floc re-growth; (2) G = 40 s^−1^ for floc growth with five cycles of a 60 s strong shear rate of G = 1000 s^−1^ followed by a weak shear rate of 5 min at G = 40 s^−1^; and (3) adding the shear rate at G = 1000 s^−1^ for 1 min and increasing pH by 0.5. In their study, the authors adopted a relative floc size (its dimension was %) with the average maximum floc size at the steady state to characterize the floc aggregation and floc breakup behaviors.

Nan et al. (2016) [20] investigated the effect of shear rate in three stages (before floc breakage, during floc breakage, and after floc breakage) on the re-growth properties of flocs using a non-intrusive optical sampling and an imaging treatment system. Kaolin clay was collected as flocculation material in a rectangular tank reactor (150 mm×150 mm) stirred with an R1342-type impeller (50 mm in diameter), and the coagulant was Polyaluminum chloride (PACl) with a basicity value of 75%. In this study, three shear procedures were provided: (1) G = 7.7, 12.8, 18.7, and 27.4 s^−1^ for floc growth → G = 113.7 s^−1^ of 1 min for floc breakage → G = 18.7 s^−1^ for floc re-growth; (2) G = 18.7 s^−1^ for floc growth → G = 86.5, 113.7, 143.2, and 175.2 s^−1^ of 1 min for floc breakage → G = 18.7 s^−1^ for floc re-growth; and (3) G = 18.7 s^−1^ for floc growth → G = 113.7 s^−1^ of 1 min for floc breakage → G = 7.7, 12.8, 18.7, and 27.4 s^−1^ for floc re-growth. Additionally, coagulations of humic acid, phosphate, or kaolin particles with alum and PACl as coagulants were investigated in Wu et al. (2019) [29]. The adopted equipment was Flocculator ZR4-2, and the floc size was measured timely using a laser diffraction instrument and a PDA 3000 in the jar test. In their experiment, two cases were extracted, including (1) G = 23 s^−1^ for alum floc formation, followed by G = 184 s^−1^ of 1 min or 10 min for floc breakage and G = 23 s^−1^ for floc regrowth and (2) effect of a change in pH (pH = 5 and 7) on the re-growth of broken alum-kaolin flocs at breakage stage (G = 23 s^−1^ → G = 184 s^−1^ of 1 min → G = 23 s^−1^).

**Figure 8:**
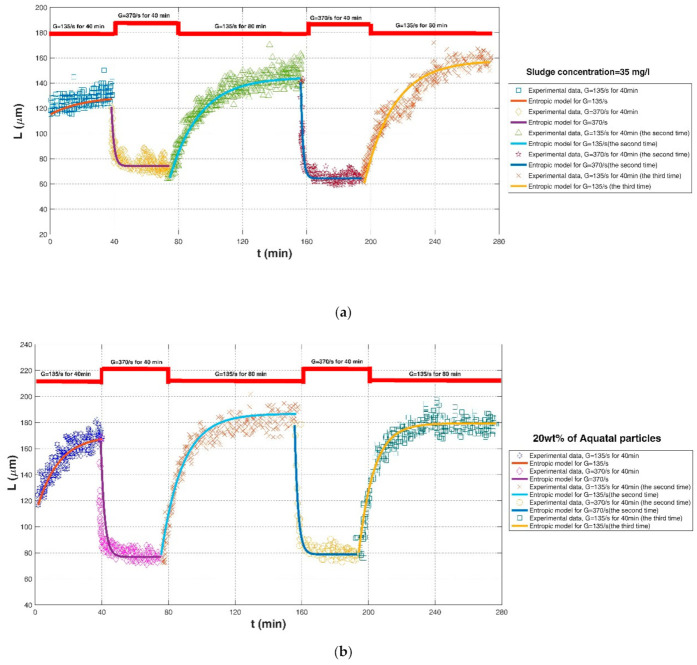
Comparison of the entropic model with experimental data points of Chaignon et al. (2002) [27]: (**a**) sludge concentration = 35 mg/L; (**b**) activated sludge spiked with 20 wt% of aquatic particles.

**Figure 9:**
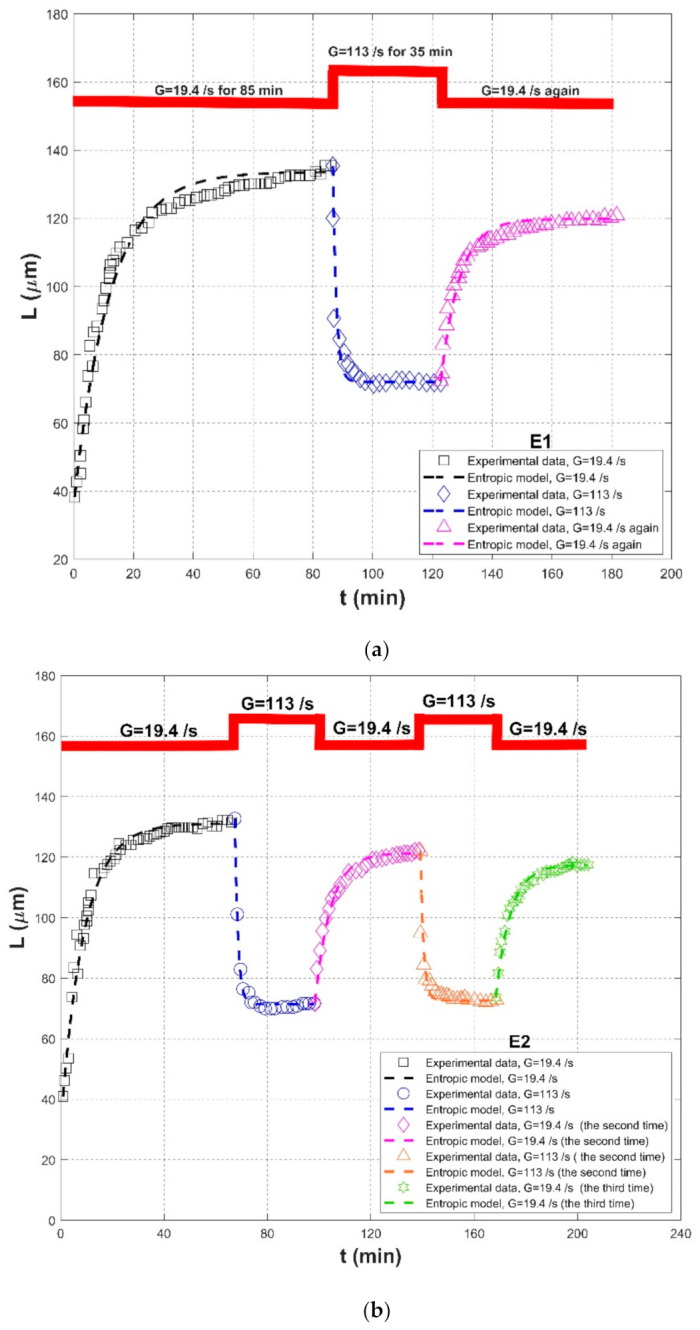
Comparison of the entropic model with experimental results of Biggs et al. (2003) [18]: (**a**) E1; (**b**) E2.

**Figure 10:**
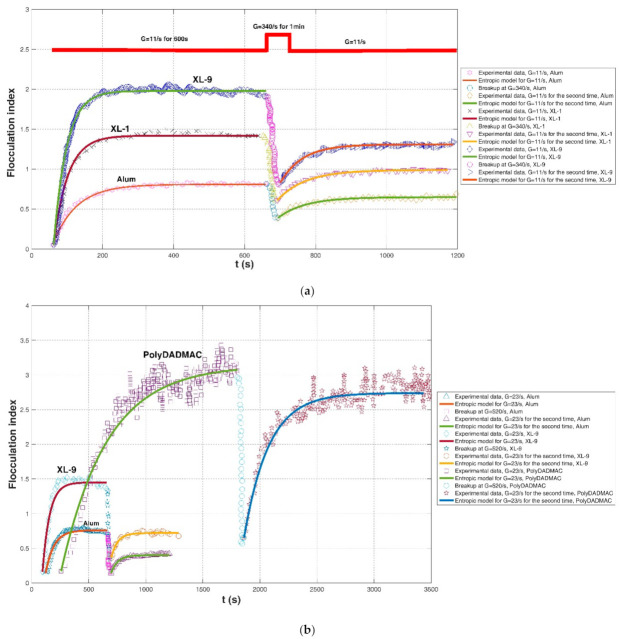
Comparison of the entropic model with experimental data points of Gregory (2004) [19]: (**a**) dosing with alum and two PACl samples; (**b**) dosing kaolin suspensions with alum, PACl, and polyDADMAC.

**Figure 11:**
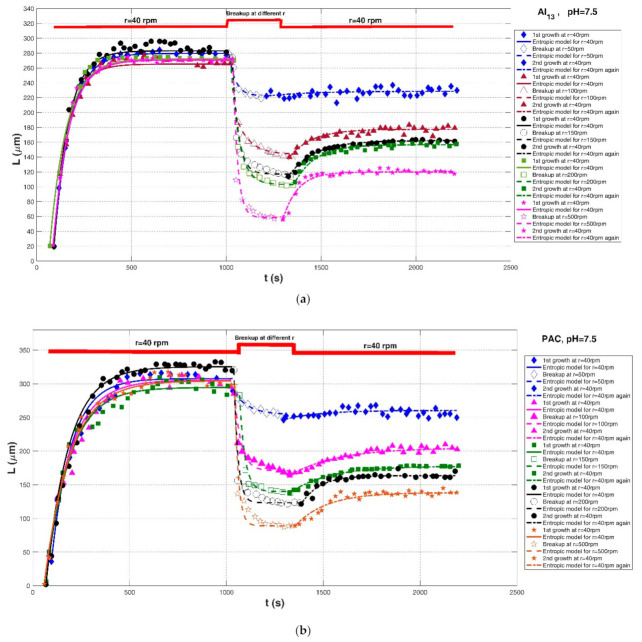

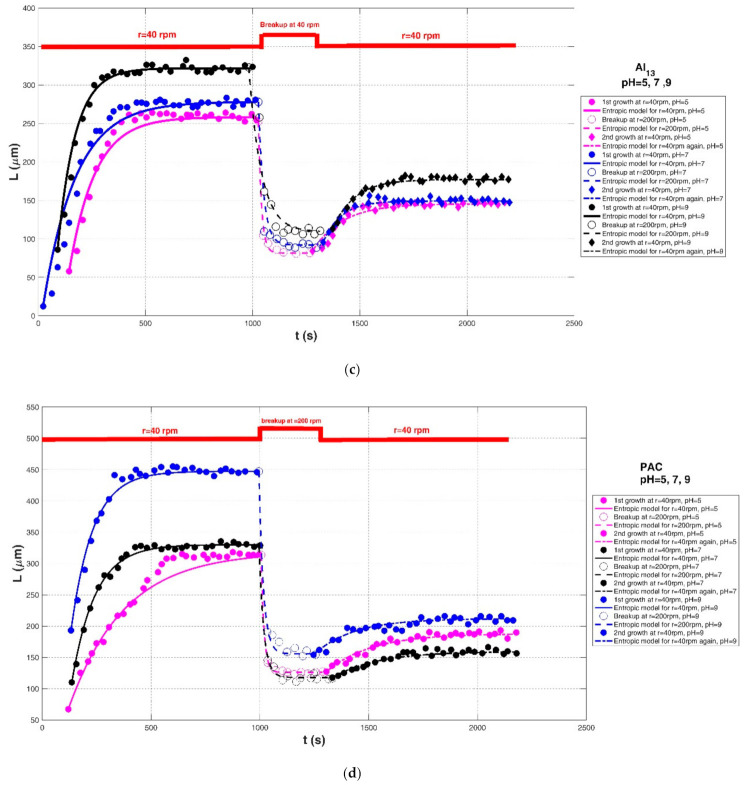
Comparison of the entropic model with experimental data points of Xu et al. (2010) [24]: (**a**) Al_13_, pH = 7.5; (**b**) PAC, pH = 7.5; (**c**) Al_13_, pH = 5, 7, 9; (**d**) PAC, pH = 5, 7, 9.

**Figure 12:**
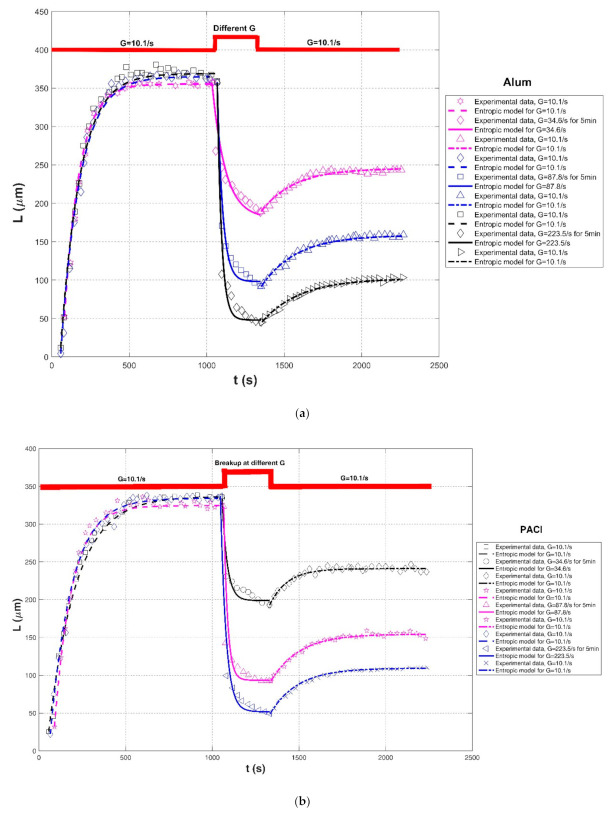

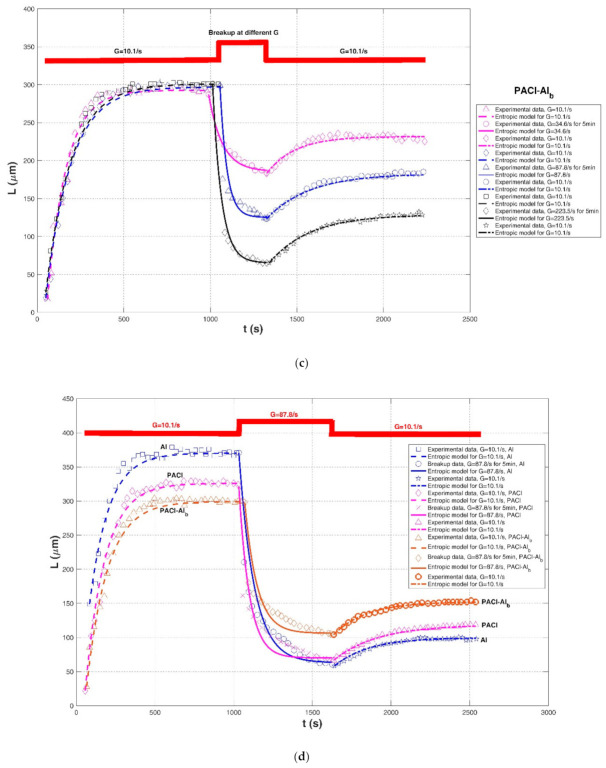
Comparison of the entropic model with experimental data points of Xu and Gao (2012) [74]: (**a**) alum; (**b**) PACl; (**c**) PACl–Al_b_ with short breakage period (5 min); (**d**) with long breakage period (10 min) by enhanced shear rates of 87.8 s^−1^.

**Figure 13:**
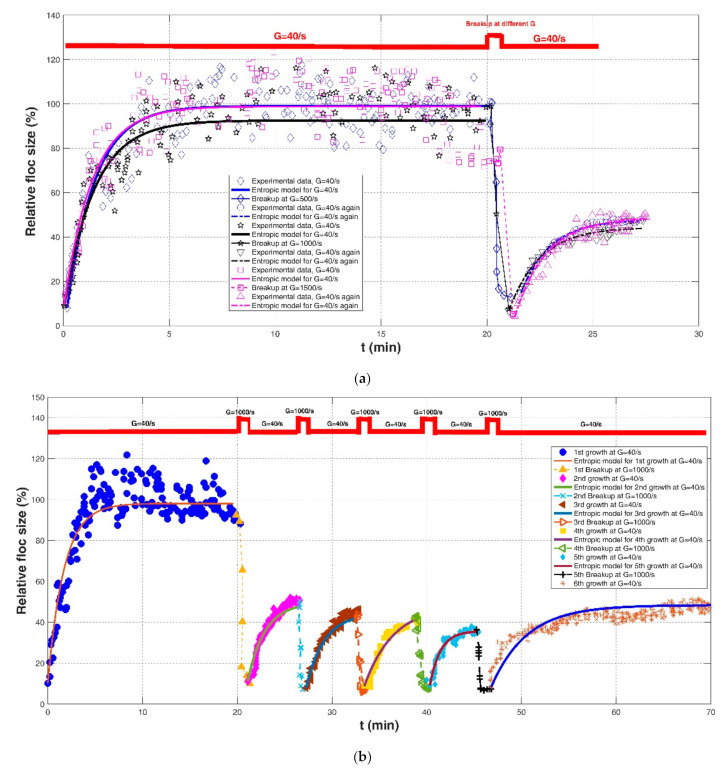

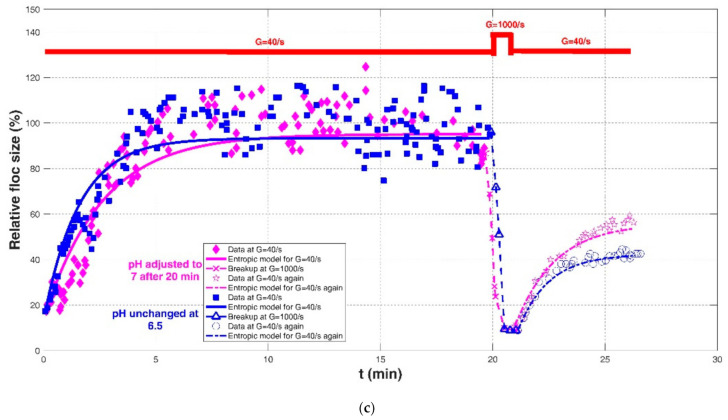
Comparison of the entropic model with experimental data points of Slavik et al. (2012) [22]: (**a**) single shear; (**b**) 5 cycles; (**c**) pH increase.

**Figure 14:**
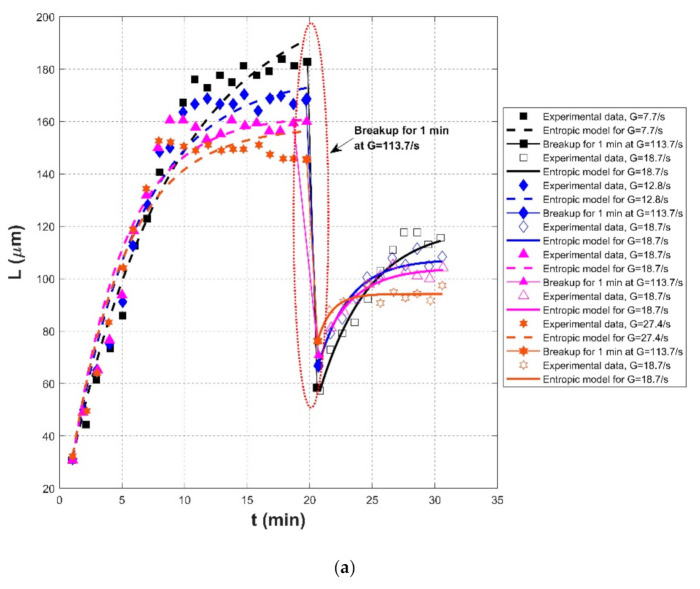

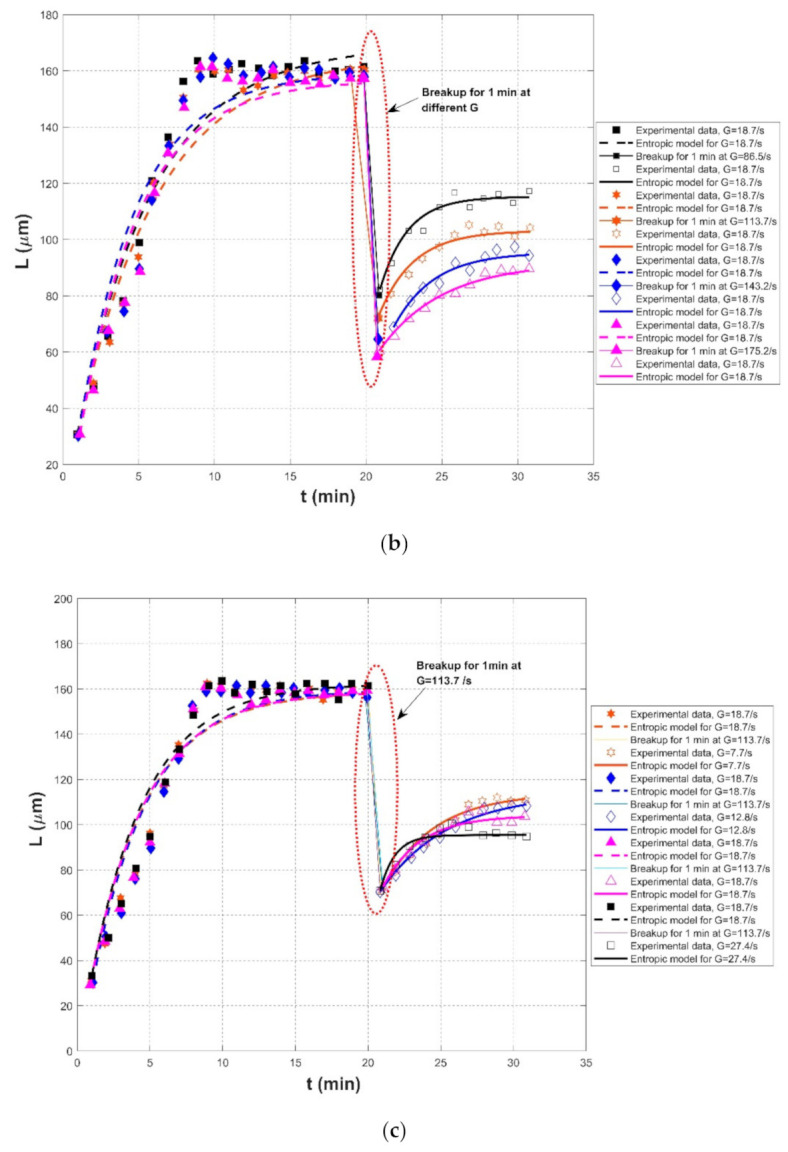
Comparison of the entropic model with Nan et al. (2016) [20]: (**a**) before floc breakage; (**b**) during floc breakage; (**c**) after floc breakage.

**Figure 15:**
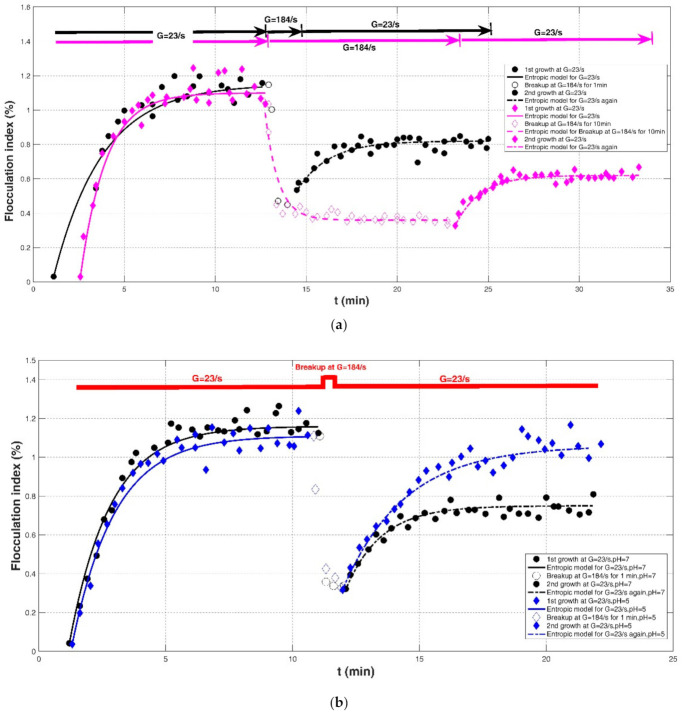
Comparison of the entropic model with Wu et al. (2019) [29]: (**a**) effect of 1 min or 10 min floc breakage on flocculation profile of alum flocs; (**b**) effect of pH changing on the re-growth of broken alum-kaolin flocs at breakage stage.

Comparisons of the proposed entropic model (Equation (11)) with experimental results of Chaignon et al. (2002) [27], Biggs et al. (2003) [18], Gregory (2004) [19], Xu et al. (2010) [24], Xu and Gao (2012) [74], Slavik et al. (2012) [22], Nan et al. (2016) [20], and Wu et al. (2019) [29] are presented in Figure 8, Figure 9, Figure 10, Figure 11, Figure 12, Figure 13, Figure 14 and Figure 15, respectively. Table 2 lists these comparison results. The second, third, fourth, and fifth columns present the flocculation material, turbulence-generated equipment, flocculation conditions, and shear rate conditions adopted in each experiment, respectively. The values for $L_{0}$ and $L_{s}$ in the sixth and seventh columns were obtained from experimental data sets. The last three columns list the calculated statistical parameter values of *R*^2^, *RE*, and *RMSE* between the proposed entropic model and each group of experimental results, as well as the value for M in the eighth column.

**Figure 16:**
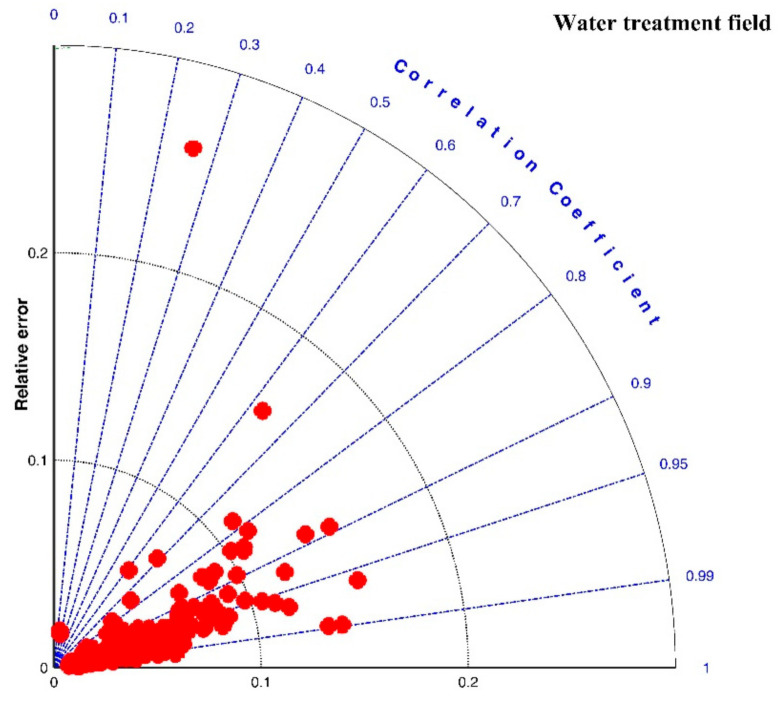
Taylor diagram of calculated correlation coefficients *R*^2^ and relative errors *RE* for experimental cases in the wastewater treatment field.

Figure 16 shows the Taylor diagram of calculated correlation coefficients and relative errors for these cases. By and large, the entropic model can agree with most experimental data points with a correlation coefficient above 0.88 and a relative error below 0.1. As listed in the last row of Table 2, the average values of *R*^2^, *RE*, and *RMSE* are 0.9301, 0.0475, and 6.6286. Three obvious large deviations in Figure 16 are from the case of G = 135 s^−1^ with sludge concentration of 35 mg/L in Chaignon et al. (2002), the case of G = 370 s^−1^ with activated sludge spiked with 20 wt% of aquatic particles in Chaignon et al. (2002), and the case of r = 40 rpm (50-rpm breakup after 40-rpm growth) in the humic acid flocculation experiment of Xu et al. (2010), as shown in F, respectively. It can be observed from Figure 8a that there was a serious data crowd in the first floc growth phase of G = 135 s^−1^, for which no flocculation models may hold; there was also no obvious trend of floc size growth from a rapid condition to a steady state. There was also a large data scatter in Figure 8b so that the entropic model did not yield a fairly satisfactory fitting effect as for other cases. In Figure 11a, the reason why the entropic model had a poor effect for the case of r = 40 rpm (50-rpm breakup after 40-rpm growth) is that there was no distinct floc growth phase directly observed from experimental data points in the authors’ paper.

As shown in Figure 9a, Figure 10, Figure 11, Figure 12, Figure 13a,c, Figure 14 and Figure 15, the flocs that formed in a low shear condition experienced an obvious breakage effect when they were abruptly subject to a strong shear rate. However, when the shear rate further went back to the original low shear condition, floc size hardly returned to the original one before that strong shear rate, indicating a limited floc recoverability. For this type of floc size variation due to a sequenced flow shear process (low shear → high shear → the original low shear), the proposed entropic model (Equation (11)) can capture well the evolution trend of floc size and have a good agreement with data points. In Figure 8 and Figure 9b, there is a cycled flow shear procedure (low shear → high shear → the original low shear → the original high shear → the original low shear), and the flocs experienced a growth phase three times and a breakage phase two times. When the shear rate went back to the original low condition after floc breakage, floc size increased to (even passing) the original one before breakage in Figure 8, indicating a strong floc recoverability; in contrast, floc size in Figure 9b did not return to the original one before floc breakup even when the shear rate became the original low condition, indicating a limited re-growth of broken flocs. In Figure 13, the flocs experienced five cycles of a low shear (G = 40 s^−1^) and high shear (G = 1000 s^−1^). Steady-state floc size indeed did not recover to the original one at flocculation time of 0 and 20 min even though the shear rate became the original low shear condition (G = 40 s^−1^) after the first-time floc breakage. However, the final floc size achieved after repeated breakage did not decrease, that is, there were no obvious final floc size variations among the second, third, fourth, fifth, and sixth floc growth phases. It can be seen that from these figures, regardless of irreversible or reversible floc growth, the entropic model can still track well the evolution trend of floc size in a cycled shear condition with high accuracy. Additionally, the entropic model was not plotted here for some floc breakage phases in Figure 10, Figure 13 and Figure 14 due to limited data points.

**Table 2:** Summary of the comparison result of the entropic model with experimental data sets regarding floc size variation subject to a varied shear rate collected in the water treatment field. The last row shows the average values of statistical errors.

References	Flocculation Experiment Condition						Fitting Effect			
	Material	Flocculation Environment	Flocculation Condition	Flocculation Period	*L*_0_ (μm)	*L_s_* (μm)	*M* (μm*min)	*R* ^2^	*RE*	*RMSE*
Chaignon et al. (2002) [27]	Activated sludge	Baffled reactor with a stirring motor	Sludge concentration = 35 mg/L	G = 135/s, for 40 min	115.30	129.64	300	0.2593	0.2593	6.5020
				G = 370/s, for 40 min breakup	121.10	74.20	75	0.6102	0.0592	6.8263
				G = 135/s, for 80 min	64.67	144.69	1500	0.9286	0.0411	6.4584
				G = 370/s, for 40 min breakup	140.51	64.46	100	0.6875	0.0725	10.7549
				G = 135/s, for 80 min	60.58	158.16	1800	0.9215	0.0651	9.0372
Chaignon et al. (2002) [27]	Activated sludge	Baffled reactor with a stirring motor	Activated sludge spiked with 20 wt% of aquatic particles	G = 135/s, for 40 min	116.56	170.20	700	0.8092	0.0361	0.0361
				G = 370/s, for 40 min breakup	165.56	76.89	200	0.6312	0.1596	28.4818
				G = 135/s, for 80 min	78.15	186.81	1400	0.9514	0.0433	7.6736
				G = 370/s, for 40 min breakup	178.15	78.81	200	0.7514	0.0492	10.3384
				G = 135/s, for 80 min	80.13	179.47	900	0.9347	0.0407	7.5324
Biggs et al. (2003) [18]	Activated sludge	Baffled batch vessel with an impeller	E1	G = 19.4/s	38.29	133.63	1150	0.9819	0.0327	4.0838
				G = 113 s	135.42	72.07	75	0.8831	0.0380	7.5874
				G = 19.4/s	72.14	119.85	300	0.9784	0.0185	2.5094
			E2	G = 19.4/s	40.92	131.08	800	0.9852	0.0205	2.9983
				G = 113 s	132.73	71.33	75	0.9946	0.0117	1.1121
				G = 19.4/s	71.69	121.37	300	0.9852	0.0155	2.2554
				G = 113 s	122.08	72.64	60	0.8453	0.0303	6.0052
				G = 19.4/s	73.59	117.47	250	0.9874	0.0119	1.6873
Gregory (2004) [19]	Kaolin	Modified jar test with different stirring rates	Alum coagulant	G = 11/s	0.06 (dimensional, hereinafter)	0.81 (dimensional, hereinafter)	50 (*s*, hereinafter)	0.9921	0.0381	0.0209
				G = 340/s, for 10 s breakup	--	-	-	-	-	-
				G = 11/s	0.39	0.65	20	0.9608	0.0153	0.0134
			XL-1: degrees of neutralization (OH/Al) = 1.9, 5.1 wt.% Al	G = 11/s	0.05	1.42	55	0.9888	0.1341	0.0693
				G = 340/s, for 10 s breakup	-	-	-	-	-	-
				G = 11/s	0.61	0.99	30	0.9835	0.0133	0.0148
			XL-9: degrees of neutralization (OH/Al) = 2.1, 4.6 wt.% Al	G = 11/s	0.05	1.98	70	0.9891	0.1407	0.1113
				G = 340/s, for 10 s breakup	-	-	-	-	-	-
				G = 11/s	0.83	1.31	30	0.9880	0.0113	0.0170
Gregory (2004) [19]	Kaolin	Modified jar test with different stirring rates	Alum coagulant	G = 23/s	0.14	0.76	45	0.9807	0.0522	0.0297
				G = 520/s, for breakup	-	-	-	-	-	-
				G = 23/s	0.14	0.40	15	0.9636	0.0359	0.0151
			XL-9: degrees of neutralization (OH/Al) = 2.1, 4.6 wt.% Al	G = 23/s	0.16	1.45	80	0.9713	0.0840	0.0833
				G = 520/s, for breakup	-	-	-	--	-	-
				G = 23/s	0.36	0.72	20	0.9720	0.0224	0.0175
			polyDADMAC coagulant (a high-charge, low-molecular-weight cationic polyelectrolyte)	G = 23/s	0.17	3.14	1200	0.9082	0.0669	0.1840
				G = 520/s, for breakup	-	--	-	-	-	-
				G = 23/s	0.65	2.74	450	0.9060	0.0448	0.1442
Xu et al. (2010) [24]	Humic acid (HA).	Jar-test apparatus with a stirrer, Al_13_ polymer at different shear rate, pH = 7.5	50-rpm breakup after 40-rpm growth	r = 40 rpm	19.63	279.74	22,000 (μm∗s, hereinafter)	0.9984	0.0116	3.1415
				r = 50 rpm	279.27	222.55	2000	0.9576	0.0111	4.6007
				r = 40 rpm	222.28	228.76	1500	0.1546	0.0181	5.0816
			100-rpm breakup after 40-rpm growth	r = 40 rpm	117.55	265.28	11,000	0.9810	0.0230	6.6168
				r = 100 rpm	186.77	143.75	3000	0.9632	0.0165	3.1873
				r = 40 rpm	140.01	177.50	4500	0.8730	0.0167	3.7291
			150-rpm breakup after 40-rpm growth	r = 40 rpm	19.63	283.18	20,000	0.9901	0.0222	7.0939
				r = 150 rpm	274.46	117.26	5000	0.9556	0.0458	10.2740
				r = 40 rpm	113.99	162.29	6500	0.9852	0.0089	1.7233
			200-rpm breakup after 40-rpm growth	r = 40 rpm	20.33	272.50	22,500	0.9909	0.0227	6.8536
				r = 200 rpm	273.10	103.04	7500	0.9822	0.0335	6.9715
				r = 40 rpm	106.43	156.72	5500	0.9487	0.0171	3.2077
			500-rpm breakup after 40-rpm growth	r = 40 rpm	21.68	270.73	21,000	0.9932	0.0138	5.3752
				r = 500 rpm	270.35	58.56	5000	0.9210	0.0911	19.5654
				r = 40 rpm	55.79	119.96	5000	0.9489	0.0255	3.6906
Xu et al. (2010) [24]	Humic acid (HA).	Jar-test apparatus with a stirrer, polyaluminum chloride polymer at different shear rate, pH = 7.5	50-rpm breakup after 40-rpm growth	r = 40 rpm	36.05	307.90	28,000	0.9735	0.0439	12.3611
				r = 50 rpm	287.22	254.27	2500	0.9447	0.0081	2.8922
				r = 40 rpm	246.78	260.54	2500	0.1824	0.0164	5.4085
			100-rpm breakup after 40-rpm growth	r = 40 rpm	45.80	305.79	32,000	0.9695	0.0502	14.1697
				r = 100 rpm	285.60	170.96	4000	0.9355	0.0338	9.2723
				r = 40 rpm	163.88	204.92	10,000	0.9456	0.0139	3.4411
			150-rpm breakup after 40-rpm growth	r = 40 rpm	5.95	294.72	35,000	0.9781	0.0443	11.6565
				r = 150 rpm	283.18	140.06	4000	0.9728	0.0295	7.4282
				r = 40 rpm	137.86	177.69	8000	0.9851	0.0079	1.7108
			200-rpm breakup after 40-rpm growth	r = 40 rpm	1.88	325.50	38,000	0.9901	0.0541	11.6801
				r = 200 rpm	320.56	123.38	4000	0.9390	0.0505	13.4764
				r = 40 rpm	121.65	163.71	4000	0.8932	0.0156	3.8415
			500-rpm breakup after 40-rpm growth	r = 40 rpm	2.69	304.50	40,000	0.9877	0.0479	10.4163
				r = 500 rpm	297.79	89.07	4000	0.9253	0.0821	16.7552
				r = 40 rpm	89.10	138.51	8000	0.9207	0.0294	4.7246
Xu et al. (2010) [24]	Humic acid (HA).	Jar-test apparatus with a stirrer, Al_13_ polymer at different shear rate	pH = 5	r = 40 rpm	57.89	258.33	24,000	0.9744	0.0431	10.7588
				r = 200 rpm	252.63	81.58	2500	0.9907	0.0351	5.7008
				r = 40 rpm	84.21	145.61	8500	0.9329	0.0317	5.5597
			pH = 7	r = 40 rpm	12.28	278.22	40,500	0.9685	0.1172	17.4689
				r = 200 rpm	278.07	92.11	4500	0.9459	0.0589	13.3823
				r = 40 rpm	95.61	149.27	4500	0.9525	0.0161	3.4224
			pH = 9	r = 40 rpm	85.96	321.78	19,500	0.9830	0.0261	9.1763
				r = 200 rpm	322.81	109.21	12,500	0.9326	0.0667	18.3096
				r = 40 rpm	113.16	177.19	6500	0.9730	0.0149	3.1652
Xu et al. (2010) [24]	Humic acid (HA).	Jar-test apparatus with a stirrer, polyaluminum chloride polymer at different shear rate	pH = 5	r = 40 rpm	67.34	318.50	64,000	0.9817	0.0393	14.2694
				r = 200 rpm	313.70	126.08	2500	0.9987	0.0122	2.0395
				r = 40 rpm	127.36	187.98	12,500	0.9567	0.0191	4.1434
			pH = 7	r = 40 rpm	110.33	330.10	23,500	0.9932	0.0143	4.9210
				r = 200 rpm	326.61	117.96	4500	0.9946	0.0287	4.3394
				r = 40 rpm	117.65	159.39	8500	0.9259	0.0208	4.1117
			pH = 9	r = 40 rpm	193.13	447.35	25,500	0.9846	0.0168	9.4848
				r = 200 rpm	447.04	155.76	6000	0.9921	0.0399	8.8524
				r = 40 rpm	154.30	211.99	10,500	0.8975	0.0209	5.3591
Xu and Gao (2012) [74]	Humic acid	Jar-test apparatus with different mixing rates	Alum coagulant	G = 10.1/s, for 15 min	52.13	355.51	30,000 (μm∗s, hereinafter)	0.9437	0.0977	24.1654
				G = 34.6/s, for 5 min breakup	357.45	180.54	15,000	0.9248	0.0496	16.5140
				G = 10.1/s, for 15 min	189.36	245.60	12,000	0.9703	0.0101	2.8881
				G = 10.1/s, for 15 min	4.30	365.05	45,000	0.9937	0.0590	10.3406
				G = 87.8/s, for 5 min breakup	358.06	98.00	9000	0.9663	0.0753	14.7623
				G = 10.1/s, for 15 min	91.40	158.38	15,000	0.9882	0.0145	2.5093
				G = 10.1/s, for 15 min	11.83	369.06	45,000	0.9913	0.0351	10.6234
				G = 223.5/s, for 5 min breakup	356.99	47.95	8500	0.9611	0.1525	18.5870
				G = 10.1/s, for 15 min	45.16	102.15	14,000	0.9911	0.0196	1.9381
Xu and Gao (2012) [74]	Humic acid	Jar-test apparatus with different mixing rates	PACl coagulant	G = 10.1/s, for 15 min	25.46	336.66	50,000	0.9924	0.0506	9.5759
				G = 34.6/s, for 5 min breakup	334.06	198.91	4000	0.9263	0.0379	11.3640
				G = 10.1/s, for 15 min	192.62	241.12	5000	0.9513	0.0097	2.8908
				G = 10.1/s, for 15 min	31.84	324.52	30,000	0.9944	0.0179	7.0992
				G = 87.8/s, for 5 min breakup	323.29	93.50	5000	0.8354	0.1023	32.0393
				G = 10.1/s, for 15 min	92.59	154.47	10,000	0.9837	0.0133	2.4118
				G = 10.1/s, for 15 min	22.22	334.26	39,000	0.9921	0.0211	7.2941
				G = 223.5/s, for 5 min breakup	337.31	51.82	9000	0.9598	0.1112	17.3637
				G = 10.1/s, for 15 min	49.59	109.48	9500	0.9921	0.0159	1.8107
Xu and Gao (2012) [74]	Humic acid	Jar-test apparatus with different mixing rates	PACl-Al_b_ coagulant	G = 10.1/s, for 15 min	16.84	292.84	30,000	0.9859	0.0480	10.0659
				G = 34.6/s, for 5 min breakup	287.37	185.79	8000	0.9736	0.0191	4.9168
				G = 10.1/s, for 15 min	183.16	231.89	8000	0.9357	0.0144	3.8579
				G = 10.1/s, for 15 min	18.95	297.14	40,000	0.9941	0.0401	7.7517
				G = 87.8/s, for 5 min breakup	298.95	125.26	7000	0.8588	0.0703	21.0826
				G = 10.1/s, for 15 min	123.16	183.03	15,000	0.9883	0.0129	2.7313
				G = 10.1/s, for 15 min	26.32	301.18	40,000	0.9941	0.0226	7.6730
				G = 223.5/s, for 5 min breakup	301.05	65.26	12,000	0.9908	0.0445	7.0965
				G = 10.1/s, for 15 min	65.26	128.87	15,000	0.9911	0.0172	2.2243
Xu and Gao (2012) [74]	Humic acid	Jar-test apparatus with different mixing rates	Alum coagulant	G = 10.1/s, for 15 min	148.96	370.24	27000	0.9770	0.0228	9.8363
				G = 87.8/s, for 10 min breakup	370.83	63.02	30,000	0.9241	0.1206	26.1279
				G = 10.1/s, for 15 min	58.33	99.66	9000	0.9813	0.0171	1.9847
			PACl coagulant	G = 10.1/s, for 15 min	21.88	326.04	38,000	0.9909	0.0239	8.1788
				G = 87.8/s, for 10 min breakup	320.83	70.05	17,000	0.8838	0.1374	23.0509
				G = 10.1/s, for 15 min	66.67	118.71	14,500	0.9949	0.0149	1.7564
			PACl-Al_b_ coagulant	G = 10.1/s, for 15 min	28.13	299.31	36,000	0.9885	0.0324	8.6583
				G = 87.8/s, for 10 min breakup	297.92	106.25	15,000	0.8735	0.0856	19.8588
				G = 10.1/s, for 15 min	104.17	152.34	10,000	0.9931	0.0073	1.2614
Slavik et al. (2012) [22]	Raw water in Saxony (Germany)	Jar test with single mixers	pH 6.5 with coagulant dosages of 0.2 mmol/L and G = 40/s for 20 min.	G = 40/s for 20 min	8.10(%, hereinafter)	99.13(%, hereinafter)	120 (%*min, hereinafter)	0.8447	0.1085	9.7307
				G = 500/s for 1 min breakup	-	-	-	-	-	-
				G = 40/s	14.76	47.79	45	0.9863	0.0212	1.1262
				G = 40/s for 20 min	9.05	92.55	120	0.8519	0.1075	10.1488
				G = 1000/s for 1 min breakup	--	-	-	-	-	-
				G = 40/s	6.67	44.39	55	0.9622	0.0562	2.0993
				G = 40/s for 20 min	9.52	98.83	120	0.7737	0.1114	11.5515
				G = 1500/s for 1 min breakup	-	-	-	--	-	-
				G = 40/s	4.29	48.71	65	0.9182	0.0734	3.2751
Slavik et al. (2012) [22]	Raw water in Saxony (Germany)	Jar test with single mixers	pH 6.5 with coagulant dosages of 0.2 mmol/L and repeatedshearing	G = 40/s for 20 min	10.22	98.12	150	0.8529	0.0837	8.7473
				G = 1000/s for 1 min breakup	-	-	-	-	-	-
				G = 40/s	11.26	49.62	70	0.9603	0.0880	3.1496
				G = 1000/s for 1 min breakup	-	-	-	-	--	
				G = 40/s	7.87	44.26	70	0.9676	0.0745	2.6660
				G = 1000/s for 1 min breakup	--	-	-	-	-	-
				G = 40/s	8.78	48.10	120	0.9529	0.1054	2.5735
				G = 1000/s for 1 min breakup	-	--	-	-	-	-
				G = 40/s	9.13	35.78	30	0.8923	0.0989	2.8444
				G = 1000/s for 1 min breakup	-	-	-	-	-	-
				G = 40/s	8.42	48.40	150	0.8595	0.0902	4.0423
Slavik et al. (2012) [22]	Raw water in Saxony (Germany)	Jar test with single mixers	pH adjusted to 7 after 20 min	G = 40/s for 20 min	17.33	95.17	200	0.8906	0.1493	11.4095
				G = 1000/s for 1 min breakup	-	-	-	-	-	-
				G = 40/s	10.89	55.94	80	0.9845	0.0633	2.8540
			pH unchanged at 6.5	G = 40/s for 20 min	17.33	93.36	120	0.8176	0.1145	0.1145
				G = 1000/s for 1 min breakup	-	-	-	-	-	-
				G = 40/s	8.91	42.35	45	0.9742	0.0368	1.7954
Nan et al. (2016) [20]	Kaolin clay	Jar test reactor with aR1342-type impeller	Effect of slow mixing before breakage	G = 7.7/s, for 20 min	30.73	210.13	1500	0.9682	0.0661	9.7669
				G = 113.7/s, for 1 min breakup	-	--	-	-	-	-
				G = 18.7/s, for 10 min	57.29	120.02	250	0.9604	0.0346	4.5132
				G = 12.8/s, for 20 min	31.25	177.60	800	0.9675	0.0603	8.8210
				G = 113.7/s, for 1 min breakup	-	-	--	-	-	-
				G = 18.7/s, for 10 min	67.19	107.42	100	0.9641	0.0214	2.7208
				G = 18.7/s, for 20 min	30.73	162.25	550	0.9556	0.0644	9.6154
				G = 113.7/s, for 1 min breakup	-	-	-	-	-	-
				G = 18.7/s, for 10 min	81.25	104.08	60	0.9289	0.0164	2.0539
				G = 27.4/s, for 20 min	32.29	157.98	550	0.9435	0.0666	9.0538
				G = 113.7/s, for 1 min breakup	-	--	-	-	-	-
				G = 18.7/s, for 10 min	76.56	94.17	20	0.8483	0.0184	2.2616
Nan et al. (2016) [20]	Kaolin clay	Jar-test reactor with a R1342-type impeller	Effect of rapid mixing during breakage	G = 18.7/s, for 20 min	30.73	169.02	700	0.9501	0.0687	10.2591
				G = 86.5/s, for 1 min breakup	-	--	-	-	-	-
				G = 18.7/s, for 10 min	82.29	115.23	60	0.9558	0.0162	2.3454
				G = 18.7/s, for 20 min	30.73	165.11	700	0.9506	0.0664	10.6025
				G = 113.7/s, for 1 min breakup	-	-	--	-	-	-
				G = 18.7/s, for 10 min	72.40	103.13	70	0.9697	0.0168	2.0119
				G = 18.7/s, for 20 min	30.21	159.27	500	0.9345	0.0762	11.4771
				G = 143.2 s, for 1 min breakup	-	-	-	-	-	-
				G = 18.7/s, for 10 min	68.75	95.44	70	0.9566	0.0158	1.9016
				G = 18.7/s, for 20 min	30.73	156.68	500	0.9509	0.0780	11.0099
				G = 175.2/s, for 1 min breakup	-	--	-	-	-	-
				G = 18.7/s, for 10 min	60.42	91.81	130	0.9880	0.0121	1.1794
Nan et al. (2016) [20]	Kaolin clay	Jar-test reactor with a R1342-type impeller	Effect of slow mixing after breakage	G = 18.7/s, for 20 min	31.25	158.75	500	0.9540	0.0798	10.7711
				G = 113.7/s, for 1 min breakup	-	--	-	-	-	-
				G = 7.7/s, for 10 min	70.83	113.94	150	0.9733	0.0210	2.4718
				G = 18.7/s, for 20 min	30.21	159.59	500	0.9461	0.0835	11.3822
				G = 113.7/s, for 1 min breakup	-	-	--	-	-	-
				G = 12.8/s, for 10 min	70.31	113.81	200	0.9914	0.0119	1.3387
				G = 18.7/s, for 20 min	29.17	158.75	500	0.9519	0.0791	10.8267
				G = 113.7/s, for 1 min breakup	-	-	-	-	-	-
				G = 18.7/s, for 10 min	72.40	103.99	80	0.9590	0.0167	2.2380
				G = 18.7/s, for 20 min	33.33	162.42	500	0.9531	0.0834	10.9332
				G = 113.7/s, for 1 min breakup	-	--	-	-	-	-
				G = 27.4/s, for 10 min	70.83	95.44	25	0.9130	0.0195	2.6405
Wu et al. (2019) [29]	Deionized (DI) water with alum and PACl_25_ as coagulants	Jar-test equipment with a stirrer	1-min breakup at 200 rpm	G = 23/s	0.03 (*%*, hereinafter)	1.15 (*%*, hereinafter)	3 (%*min, hereinafter)	0.9399	0.0561	0.0691
				G = 184/s for 1 min breakup	--	-	-	-	-	-
				G = 23/s	0.53	0.82	0.5	0.7810	0.0359	0.0379
			10-min breakup at 200 rpm	G = 23/s	0.03	1.10	1.5	0.9534	0.0606	0.0685
				G = 184/s for 10 min breakup	1.05	0.36	0.5	0.9056	0.0683	0.0575
				G = 23/s	0.33	0.62	0.4	0.9006	0.0328	0.0250
Wu et al. (2019) [29]	Deionized (DI) water with alum and PACl_25_ as coagulants	Jar-test equipment with a stirrer	pH = 7	G = 23/s	0.04	1.16	1.8	0.9783	0.0545	0.0535
				G = 184/s for 1 min breakup	-	-	-	-	--	-
				G = 23/s	0.32	0.75	0.6	0.9269	0.0359	0.0315
			pH = 5	G = 23/s	0.04	1.11	1.8	0.9648	0.0534	0.0573
				G = 184/s for 10 min breakup	-	-	-	-	-	-
				G = 23/s	0.32	1.06	1.8	0.9506	0.0420	0.0505
**In average**								**0.9301**	**0.0475**	**6.6286**

#### 3.2.3. Colloidal Science Field

Two experimental data sets were collected from the literature in the colloidal science field to investigate the effect of varied shear rates on the floc size in this study, including Spicer et al. (1998) [23] and Wu and Ven (2009).

In the laboratory experiment of Spicer et al. (1998) [23], the impact of a varied shear history on the evolution of the polystyrene-alum floc size, density, and structure was investigated. Flocculation material was suspensions of monodisperse polystyrene particles, and the flocculation apparatus was 2.8-L baffled tank with a radial flow Lightnin R100 impeller. The particle volume fraction was 1.4×10^−5^. Floc size distribution and floc structure were estimated by small-angle light scattering measurements via a Malvern Mastersizer E. In this experiment, two kinds of shear procedures were involved, including (1) cycled-shear flocculation: G = 50 s^−1^ for floc formation → G = 100, 300 and 500 s^−1^ of 1 min for floc breakage → G = 50 s^−1^ for floc re-growth; and (2) tapered-shear flocculation: G = 300 s^−1^ of 15 min for floc formation → G = 200 s^−1^ of 15 min for floc formation → G = 100 s^−1^ of 15 min for floc formation → G = 50 s^−1^ of 15 min for floc formation. In the experimental study of Wu and Ven (2009), coagulation materials were separated from thermomechanical pulp particles (TMP) with a size range of 1–20 μm, and they were allowed to flocculate in a beaker. The flocculation degree was measured by a photometric dispersion analysis (PDA) for small fines and by focused beam reflectance measurements (FBRM) for a mixture of fines and fibers in distilled water or salt water. In this experiment, the effects of salt and poly (ethylene oxide) (PEO) entanglement on the flocculation of small particles were investigated. Five NaCl concentrations, 0 (no salt), 10, 20, 50, and 100 Nm, and three classes of stirring speeds, 100 rpm for floc formation, 450 rpm for floc breakage, and 100 rpm for floc re-growth, were adopted in the experiment, respectively. Since the ratio of light transmittance value to mean transmittance value was only recorded, the flocculation index was adopted to represent the relative floc size during coagulation in their study.

**Figure 17:**
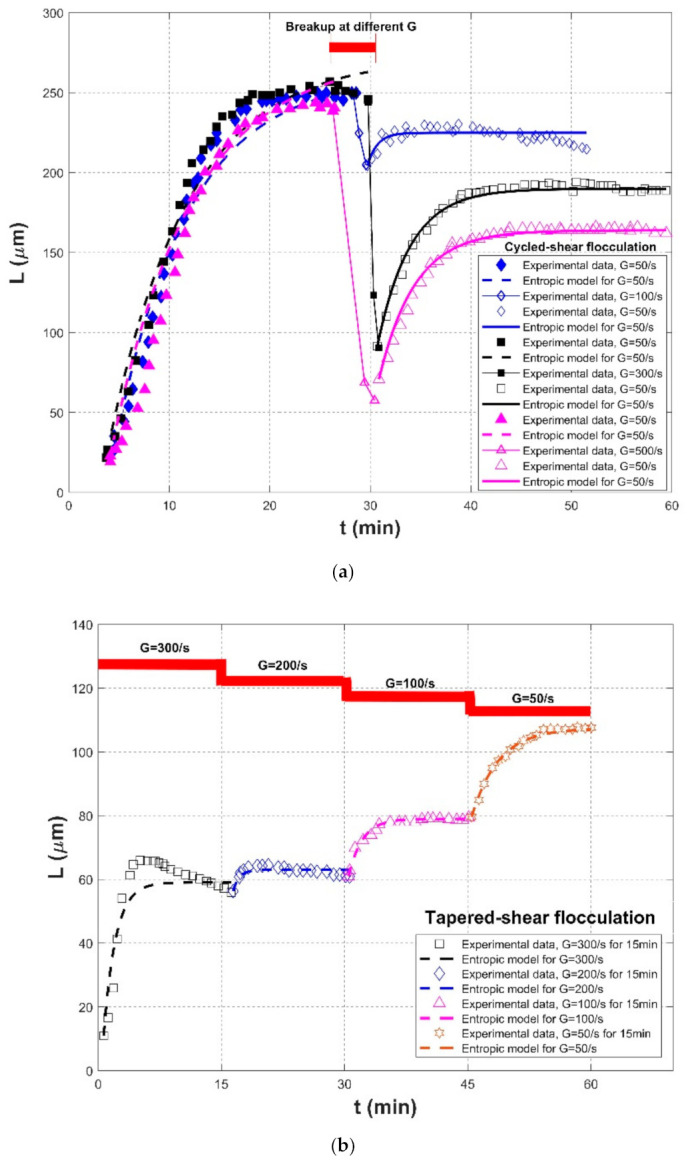
Comparison of the entropic model with experimental results of Spicer et al. (1998) [23]: (**a**) effect of cycled-shear; (**b**) effect of tapered-shear.

**Figure 18:**
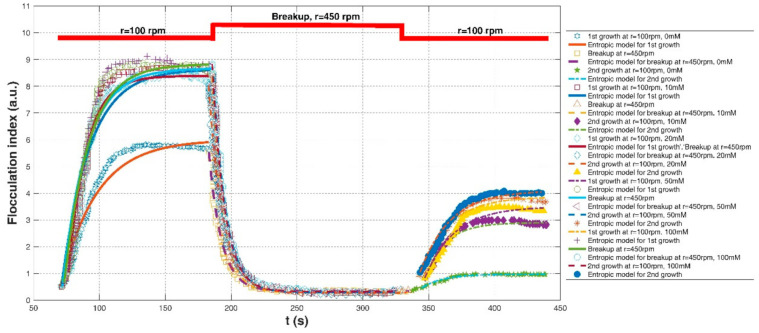
Comparison of the entropic model with experimental data of Wu and Ven (2009).

Figure 17 and Figure 18 compare the entropic model (Equation (11)) with experimental data points of Spicer et al. (1998) [23] and Wu and Ven (2009), respectively. Table 3 presents these comparison results. Similar to Table 1 and Table 2, the second through seventh columns of this table list the information regarding flocculation material, flocculation apparatus, flocculation condition, the adopted shear rates, and the values for $L_{0}$ and $L_{s}$, respectively. The performance of the entropic model for each experimental data set in terms of three statistical parameters, *R*^2^, *RE*, and *RMSE*, is provided in the last three columns, as well as the fitting values M in the eighth column.

**Figure 19:**
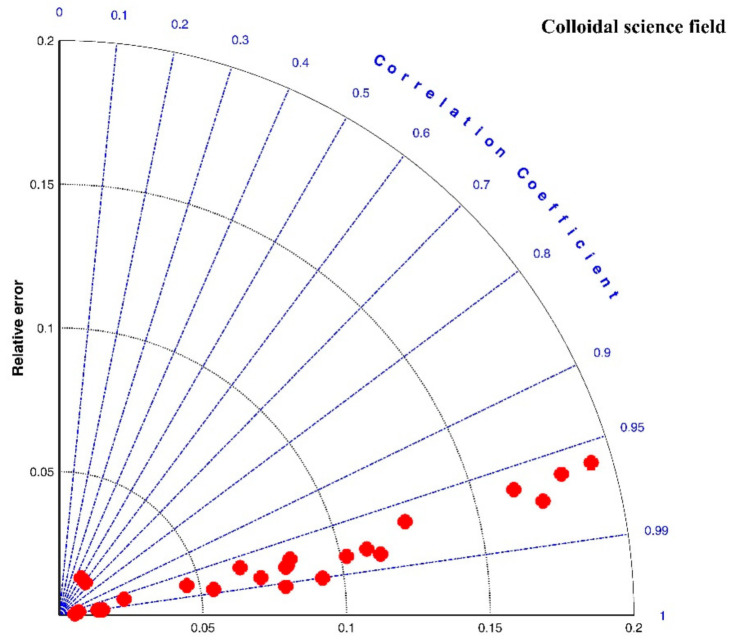
Taylor diagram of calculated correlation coefficients *R*^2^ and relative errors *RE* for experimental cases in the colloidal science field.

Figure 19 presents the Taylor diagram of the calculated correlation coefficients and relative errors for these experimental cases. Compared with Figure 7 and Figure 16, it is clear that the proposed entropic model can have a stronger correlation with experimental data sets but with a larger relative error. In general, as shown in the last row of Table 3, the average values of *R*^2^, *RE*, and *RMSE* are 0.9419, 0.0805, and 2.7264. Four large relative errors came from the third floc growth case of G = 500 s^−1^ in the Cycled-shear flocculation experiment of Spicer et al. (1998), three floc growth phases of r = 100 rpm (NaCl concentrations were 10, 50, and 100 mM in the flocculation experiment of Wu and Ven (2009), as illustrated by Figure 17a and Figure 18, respectively. In Figure 17a, as denoted by the orange color, floc size slowly increases at the first G = 500 s^−1^, whereas the entropic model overestimated these data points since it predicted a rapid floc growth phase at the initial flocculation stage. It can be observed from Figure 18 that there were some deviations between the modeled and the measured data, especially at the first floc growth period of r = 100 rpm when NaCl concentrations were 10, 50, and 100 mM. The measured floc size seemed to approach its steady state earlier than that predicted by the model, which led to an error between the predicted values and the measured ones.

In Figure 17a, polystyrene-alum floc size increased until a steady state was reached at G = 50 s^−1^; however, when a stronger shear was applied into the flocculation system, a strong floc breakage was obvious. As the shear rate went back to G = 50 s^−1^, floc size did not recover to the original level. The entropic model can depict well this pattern of irreversible floc growth. In Figure 17b, a piecewise decrease in the shear rate (G = 300 → 200 → 100 → 50 s^−1^) for 15 min allowed the flocs to have enough time to grow toward different equilibrium conditions. For this type of floc size variation in a piecewise decreasing shear condition, the entropic model can exhibit good performance. However, it is also notable that there was a deviation between the model and the data points at G = 300 s^−1^. This is because there was a floc restructuring during flocculation, as pointed out by Spicer et al. (1998) [23], for which the proposed entropy-based model did not hold. In Figure 18, the entropic model could trace well the entire process of floc growth at a low shear rate, floc breakage at a strong shear condition, and floc re-growth at a returning low shear environment for all cases.

**Table 3:** Summary of the comparison result of the entropic model with experimental data regarding floc size variation subject to a varied shear rate collected in the colloidal science field. The last row shows the average values of statistical errors.

References	Flocculation Experiment Condition						Fitting Effect			
	Material	Flocculation Environment	Flocculation Condition	Flocculation Period	*L*_0_ (um)	*L_s_* (um)	*M* (um * min)	*R* ^2^	*RE*	*RMSE*
Spicer et al. (1998) [23]	Polystyrene-alum floc	Stirred tank with a Rushton impeller	Tapered-shear flocculation	G = 300/s, for 15 min	10.93	59.03	70	0.9064	0.1043	5.8630
				G = 200/s, for 15 min	56.30	63	5	0.6229	0.0146	1.0965
				G = 100/s, for 15 min	60.93	78.98	30	0.9834	0.0069	0.7520
				G = 50/s, for 15 min	79.63	107.35	90	0.9947	0.0055	0.7335
			Cycled-shear flocculation	G = 50/s, for 30 min	26.66	260.42	1700	0.9782	0.0806	12.4689
				G = 100/s, for 1 min	-	-	-	-	--	
				G = 50/s, for 30 min	204.28	224.91	20	0.5020	0.0151	4.2064
				G = 50/s, for 30 min	21.81	272.52	2000	0.9768	0.0812	12.9654
				G = 300/s, for 1 min	-	-	--	-	--	-
				G = 50/s, for 30 min	91.49	189.90	320	0.9907	0.0136	2.7977
				G = 50/s, for 30 min	22.80	272.52	2000	0.9732	0.1730	19.0067
				G = 500/s, for 1 min	-	-	-	-	--	-
				G = 50/s, for 30 min	70.73	163.88	330	0.9920	0.0152	2.8624
Wu and Ven (2009) [25]	Thermomechanical pulp (TMP) particles at various CPR (carboxylated phenolic resin) –PEO (poly(ethylene oxide)) ratios	A beaker with a stirrer (the stirring speed from 100 to 450 rpm)	NaCl concentration: 0 mM	r = 100 rpm	0.52 (a.u. hereinafter)	6.00 (a.u. hereinafter)	150 (a.u.*s, hereinafter)	0.9653	0.1247	0.3793
				r = 450 rpm for breakup	5.54	0.34;	50	0.9715	0.0827	0.2920
				r = 100 rpm	0.46	0.97	8	0.9702	0.0233	0.0267
			NaCl concentration: 10 mM	r = 100 rpm	0.51	8.64	170	0.9626	0.1816	0.7183
				r = 450 rpm for breakup	8.37	0.29	70	0.9775	0.1095	0.4264
				r = 100 rpm	0.85	2.9	32	0.9737	0.0456	0.1249
			NaCl concentration: 20 mM	r = 100 rpm	1.04	8.39	100	0.9823	0.1139	0.5077
				r = 450 rpm for breakup	8.04	0.27	85	0.9902	0.0926	0.2762
				r = 100 rpm	0.83	3.5	60	0.9670	0.0651	0.2028
			NaCl concentration: 50 mM	r = 100 rpm	0.53	8.67	150	0.9612	0.1926	0.7468
				r = 450 rpm for breakup	8.35	0.29	78	0.9795	0.1021	0.4129
				r = 100 rpm	0.88	3.87	65	0.9830	0.0714	0.1773
			NaCl concentration: 100 mM	r = 100 rpm	0.64	8.83	150	0.9639	0.1641	0.6959
				r = 450 rpm for breakup	8.83	0.32	80	0.9919	0.0795	0.2579
				r = 100 rpm	1.04	4.10	70	0.9862	0.0545	0.1632
**In average**								**0.9419**	**0.0805**	**2.7264**

## 4. Discussion

### 4.1. Parameterization of $L_{s}$ and M

Two important parameters remain to be determined in Equations (10)–(12): $L_{s}$ and M. It can be seen from Table 1, Table 2 and Table 3 that they were varied in orders of magnitude, which implies that these parameters might be dependent on different flocculation environments (i.e., flocculation material, flocculant, particle concentration) and flow turbulent conditions, as well as flocculation recoverability (partially or completely). This study focused on the effect of varied flow shear rate on the flocculation process. Furthermore, since there are few laboratory flocculation studies concerning a continuously varied flow shear environment (except for Spicer et al. (1998) [23]), as presented in Section 3, and the units for M are sometimes different in Table 1, Table 2 and Table 3, it seemed difficult to compare the estimated values of $L_{s}$ and M for specific water/sediment, wastewater, and colloidal systems. However, for a specific particle flocculation system, if a continuously varied flow shear environment is only applied while keeping other conditions fixed, it is feasible to find the single dependencies of $L_{s}$ and M on the flow shear rate. For example, Spicer et al. (1998) [23] measured polystyrene alum floc size variations when the flow shear rate G piecewise decreased from 300, 200, and 100 to 50 s^−1^, as presented by Figure 17b. This case could facilitate us to parameterize $L_{s}$ and M, as shown as follows.

Some flocculation experiments have shown that the characteristic size of the floc population at the steady state, often denoted by median size $L_{s}$ or maximum floc size $L_{f,\max}$, decays monotonically with an increasing shear rate G. A power function has always been adopted to describe this relationship [21,23,26,49,78,79,80]:

(13) $$L_{s}\mathrm{or}\;L_{f,\max}=c*{\left(G\right)}^{-1*\gamma }$$

where c and γ are two empirical dimensional coefficients (c > 0, and γ > 0). The parameter c represents the floc strength subject to a flow shear, which is dependent on the method adopted to measure the floc size [3,42]. The γ is the floc size exponent, which depends on the hydrodynamic mechanism of floc breakup. When floc size is smaller than the smallest eddy scale of the turbulent flow (i.e., the Kolmogorov microscale length equal to ${\left(\nu^{3}/\varepsilon \right)}^{1/4}$), some particles or fragile flocs on the surface of the “mother” floc are eroded, and the floc undergoes the breakage effect in terms of surface erosion. For those flocs larger than the Kolmogorov microscale, they experience a large-scale fragmentation due to a large fluctuation of the turbulent flow [3,42]. As summarized by Zhu et al. (2010) [81] and Rau et al. (2018) [82] for some experimental data sets of the steady-state floc size–flow shear rate relationship collected from the literature, γ has a range of 0.11–0.75, whereas c has different values for different types of flocs and flocculants.

Regarding the proposed parameter, i.e., the maximum capacity for floc size increase (or decay) M, Zhu (2018) estimated its values with various flow shear rates G by fitting Equation (1) to experimental data sets from Oles (1992), Serra et al. (1997), Serra and Casamitjana (1998), Biggs and Lant (2000), and Colomer et al. (2005). There exists an empirical power function similar to Equation (13) to describe the dependence of M on G: $M=10^{6}G^{-0.844}$ with a high correlation coefficient above 0.93, indicating an important role of floc breakage due to an increasing flow shear rate in the flocculation process. Thus, the following expression can be adopted to describe M as a function of G in this study:

(14) $$M=a*{\left(G\right)}^{-1*b}$$

where a and b are two empirical coefficients (a > 0, and b > 0).

**Figure 20:**
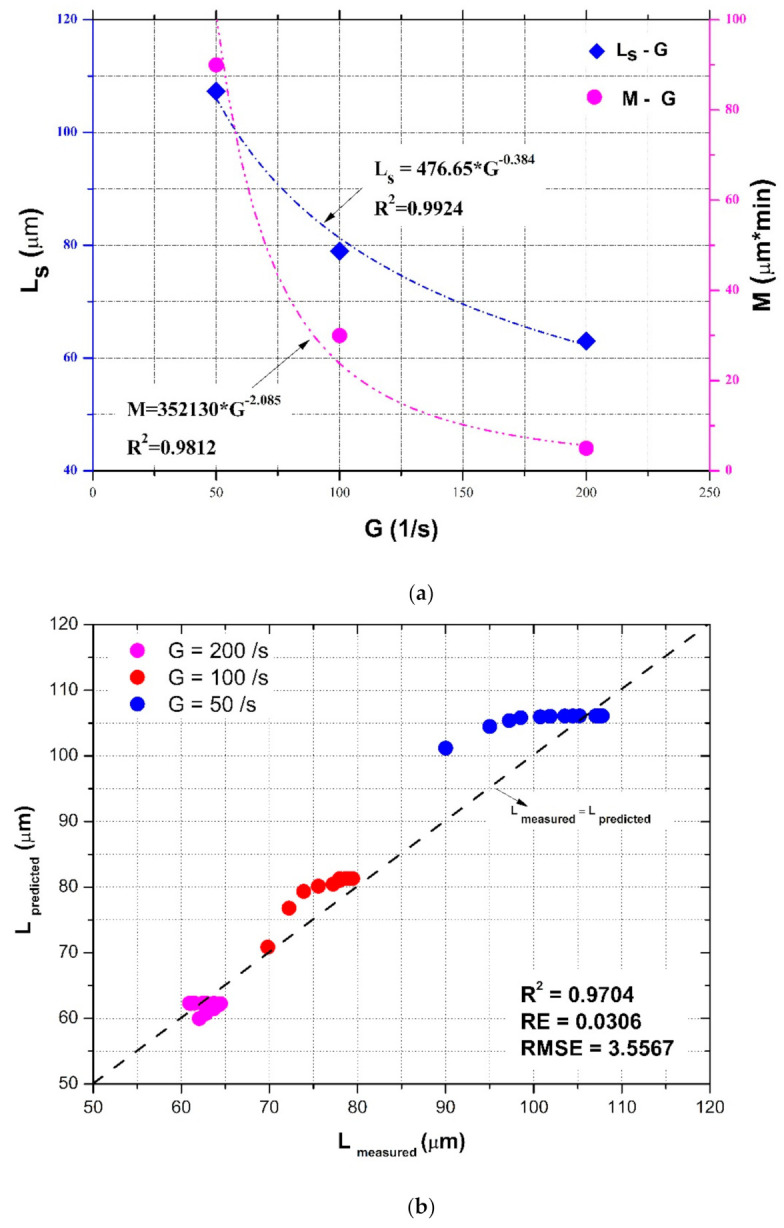
(**a**) Dependences of the steady-state floc size and the capacity parameter in the entropic model on the shear rate based on experimental data of Spicer et al. (1998) [23]; (**b**) comparison of the calibrated entropic model with measured floc size data at G = 200, 100, and 50 s^−1^ from Spicer et al. (1998) [23].

For the floc size evolution in a piecewise decaying (or enhancing) shear condition, floc size could monotonically increase (or decrease) toward an equilibrium, provided flocculation time is enough at each constant shear rate period. In this case, Equation (13) could be used to estimate the steady-state floc size, i.e., $L_{s}$ in Equations (10)–(12), as also presented by Keyvani and Strom (2014) [28] and Slavik et al. (2012) [22], who reported that the final floc size was only dependent on the turbulent shear rate. Equation (14) can also be adopted to characterize the parameter M as a function of the shear rate G in Equations (10)–(12). Take the abovementioned experimental data sets in Spicer et al. (1998) [23] as an example, as shown by Figure 17b, except the case of G = 300 s^−1^ (where floc restructuring plays a role and the model does not hold as already mentioned). In Figure 17b, the steady-state floc size $L_{s}$ can be found to increase with a decaying shear rate G. An increasing trend of the fitted parameter M with the shear rate G can be also found in Table 3. Figure 20a shows the dependences of $L_{s}$ and M on G using Equations (13) and (14), respectively. In this figure, two exponential relations, $L_{s}=476.65*G^{-0.384}$ and $M=352130*G^{-2.085}$, can have a good fitting effect with calculated data points (correlation coefficients *R*^2^ reach 0.9924 and 0.9812, respectively). Substituting them into Equation (11) can calibrate the proposed entropic model, and a comparison of floc size inverted by the calibrated entropic model with measured floc size data at G = 200, 100, and 50 s^−1^ from Spicer et al. (1998) [23] is presented in Figure 20b. It can be seen from this figure that, in general, a good agreement between the measured values and the estimated values is noticeable with a high *R*^2^ value of 0.9704, low *RE* value of 0.0306, and a low *RMSE* value of 3.5567. The discrepancy that the model overestimated the measured data for the cases of G = 50 and 100 s^−1^ may have been due to underestimated M values by the model.

It needs to be pointed out that Equations (13) and (14) solely characterize the dependencies of $L_{s}$ and M on G. Other factors, such as particle concentration and suspended solid size related to water quality variation, will also affect the steady-state floc size $L_{s}$, as revealed by some laboratory experiments. However, these dependency analyses were not performed in this study due to few relevant complete data sets from laboratory-controlled flocculation experiments and/or in situ observation from literature. Performing complete flocculation experiments subject to multiple factors and/or field floc size observations in a complicated hydrodynamic environment is worthy of further investigation in future studies.

### 4.2. Sensitivity of the Model to Four Empirical Parameters

**Figure 21:**
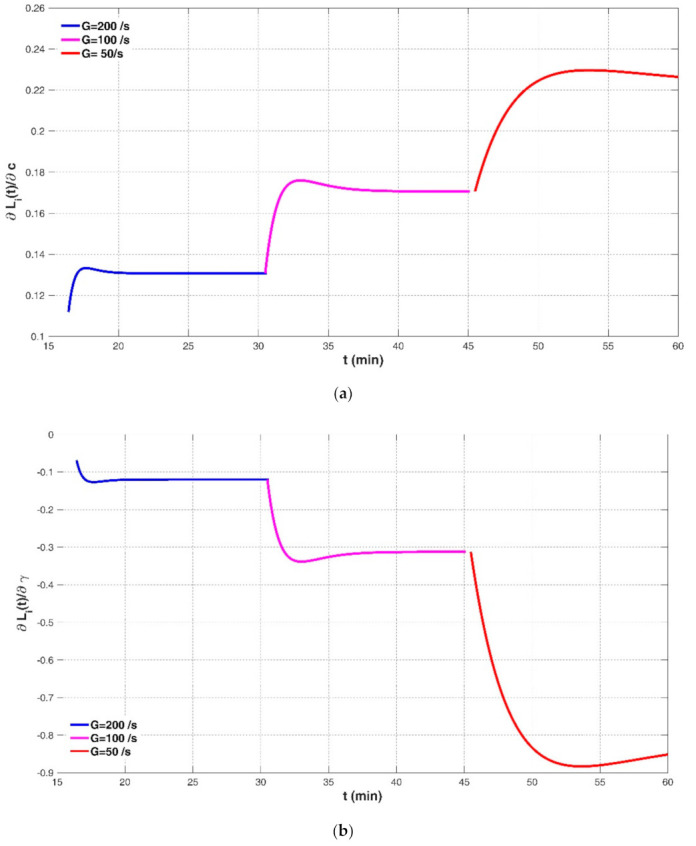

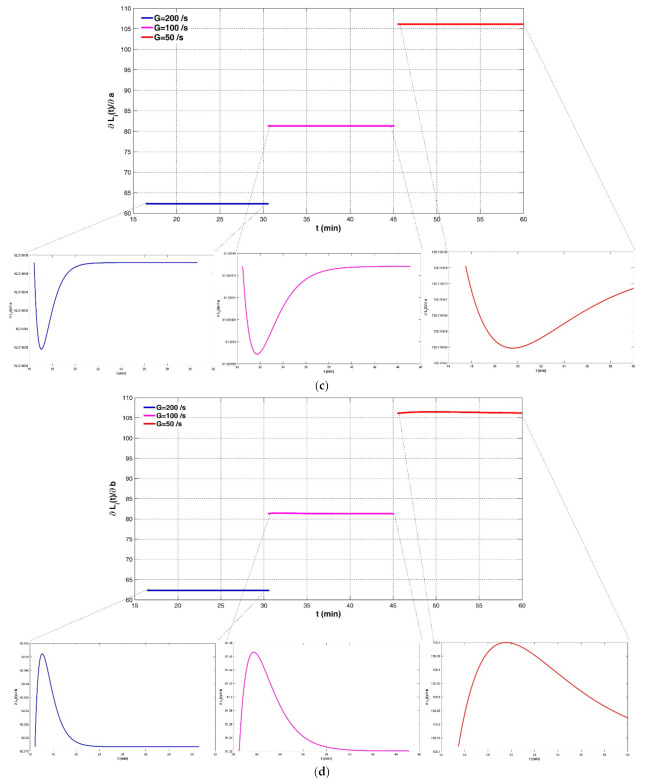
Sensitivity of the entropic flocculation model to four empirical parameters, including (**a**) c, (**b**) γ, (**c**) a, and (**d**) b for the tapered-shear flocculation cases of G = 200, 100, and 50 s^−1^ in Spicer et al. (1998) [23].

Furthermore, introducing Equations (13) and (14) into Equation (11) can yield an entropic flocculation model,

(15) $$L_{i}\left(t\right)=c*G_{i}^{-\gamma }-\left(c*G_{i}^{-\gamma }-c*G_{i-1}^{-\gamma }\right)\exp\left[-\frac{\left|c*G_{i}^{-\gamma }-c*G_{i-1}^{-\gamma }\right|}{a*G_{i}^{-b}}\left(t-t_{i-1}\right)\right]$$

which can be adopted to predict the floc size evolution in a piecewise varied shear provided flocculation time is enough to allow the floc size growth to reach the steady state at each constant shear rate period. It will be then interesting to determine the sensitivity of this model to its parameters. To this end, derivatives of $L_{i}\left(t\right)$ with respect to four input parameters a, b, c, and γ are written:

$$\begin{array}{l} \frac{\partial L_{i}\left(t\right)}{\partial c}=G_{i}^{-\gamma }-\left(G_{i}^{-\gamma }-G_{i-1}^{-\gamma }\right)f\left(t\right)-\left(c*G_{i}^{-\gamma }-c*G_{i-1}^{-\gamma }\right)f\left(t\right)\\ {}*\left[-\frac{\left(G_{i}^{-\gamma }-G_{i-1}^{-\gamma }\right)}{a*G_{i}^{-b}}\left(t-t_{i-1}\right)\right] \end{array}$$

$$\begin{array}{l} \frac{\partial L_{i}\left(t\right)}{\partial \gamma }=c*\left(-\gamma \right)*G_{i}^{-\gamma -1}-\left[c*\left(-\gamma \right)*G_{i}^{-\gamma -1}-c*\left(-\gamma \right)*G_{i-1}^{-\gamma -1}\right]f\left(t\right)\\ -\left(c*G_{i}^{-\gamma }-c*G_{i-1}^{-\gamma }\right)f\left(t\right)\left\{-\frac{\left[c*\left(-\gamma \right)*G_{i}^{-\gamma -1}-c*\left(-\gamma \right)*G_{i-1}^{-\gamma -1}\right]}{a*G_{i}^{-b}}\left(t-t_{i-1}\right)\right\} \end{array}$$

$$\frac{\partial L_{i}\left(t\right)}{\partial a}=c*G_{i}^{-\gamma }-\left(c*G_{i}^{-\gamma }-c*G_{i-1}^{-\gamma }\right)f\left(t\right)\left[\frac{\left(c*G_{i}^{-\gamma }-c*G_{i-1}^{-\gamma }\right)}{a^{2}*G_{i}^{-b}}\left(t-t_{i-1}\right)\right]$$

(16) $$\begin{array}{l} \frac{\partial L_{i}\left(t\right)}{\partial b}=c*G_{i}^{-\gamma }-\left(c*G_{i}^{-\gamma }-c*G_{i-1}^{-\gamma }\right)f\left(t\right)\\ {}*\left[-\frac{\left(c*G_{i}^{-\gamma }-c*G_{i-1}^{-\gamma }\right)}{a}b\left(t-t_{i-1}\right)G_{i}^{b-1}\right] \end{array}$$

where $f\left(t\right)=\exp\left[-\frac{\left(c*G_{i}^{-\gamma }-c*G_{i-1}^{-\gamma }\right)}{a*G_{i}^{-b}}\left(t-t_{i-1}\right)\right]$ and $c*G_{i}^{-\gamma }>c*G_{i-1}^{-\gamma }$ for convenience. Each derivative of $L_{i}\left(t\right)$, with respect to the mentioned parameter while fixing other parameters, can be regarded to be a sensitivity coefficient [70] and it can help evaluate the effect or significance of each parameter on the floc size evolution. As an example, Figure 21a–d shows the calculated values of sensitivity coefficients for the tapered-shear flocculation cases (G = 200, 100, and 50 s^−1^) of Spicer et al. (1998) [23] using Equations (16), respectively. It shows that the sensitivity coefficients to a and b exhibit a discontinuous and piecewise increase with flocculation time t, and they are much larger than the sensitivity coefficients to c and γ. The sensitivity to c increases piecewise as flocculation time proceeds, while the sensitivity to γ decreases piecewise.

Specifically, a simple flocculation model at a constant shear rate can be obtained by introducing Equations (13) and (14) into Equation (10) and adopting the initial condition: $L=L_{0}$ at $t=t_{0}=0$,

(17) $$L\left(t\right)=cG^{-\gamma }-\left(cG^{-\gamma }-L_{0}\right)\exp\left(-\frac{\left|cG^{-\gamma }-L_{0}\right|}{aG^{-b}}t\right)$$

**Figure 22:**
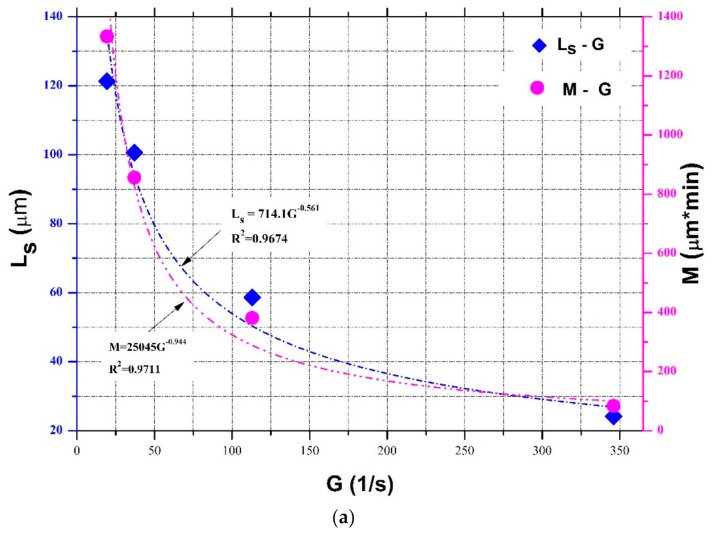

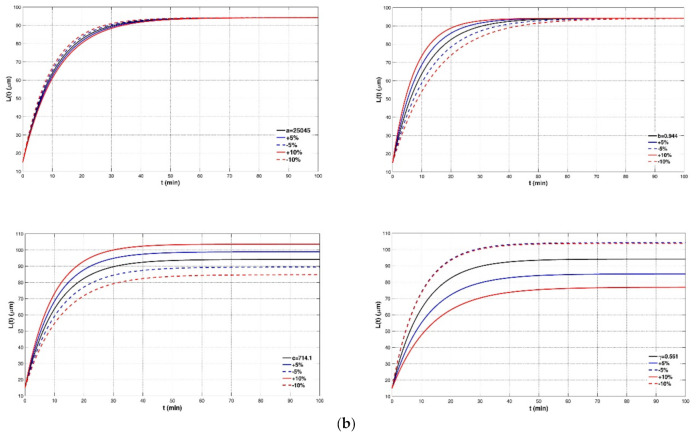
(**a**) Dependences of the steady-state floc size $L_{s}$ and the capacity parameter M on the shear rate G from Biggs and Lant (2000); (**b**) qualitative impacts of all of four parameters on the flow growth curve, including a (left, upper panel), b (right, upper panel), c (left, lower panel), and γ (right, lower panel).

Take the flocculation experimental results of activated sludge at four different kinds of constant shear rates: G = 19.4, 37, 113, and 346 s^−1^ by Biggs and Lant (2000) as an example. In their study, flocculation started not from the primary particle but $L_{0}$ = 15 μm. Steady-state floc size $L_{s}$ as a function of shear rate G is plotted in Figure 22a, where the dependence of the fitting parameter M on the shear rate G calculated by Zhu (2018) is also plotted. From it, there are a = 25045, b = 0.944,c = 714.1, and γ = 0.561. Figure 22b shows the influence of the variation of each parameter (±5%, ±10%) on the floc growth curve while fixing other parameters at G = 37 s^−1^ in Biggs and Lant (2000). It can be seen that increasing c or decreasing γ leads to an increase of the steady-state floc size, whereas either increasing a or decreasing b makes the floc growth curve become much gentler toward the steady state. This qualitative result could help fit Equation (17) with experimental data points at a constant shear rate.

### 4.3. Effect of Repeated Cycles of a Low and High Shear Rate on the Floc Size Growth

**Figure 23:**
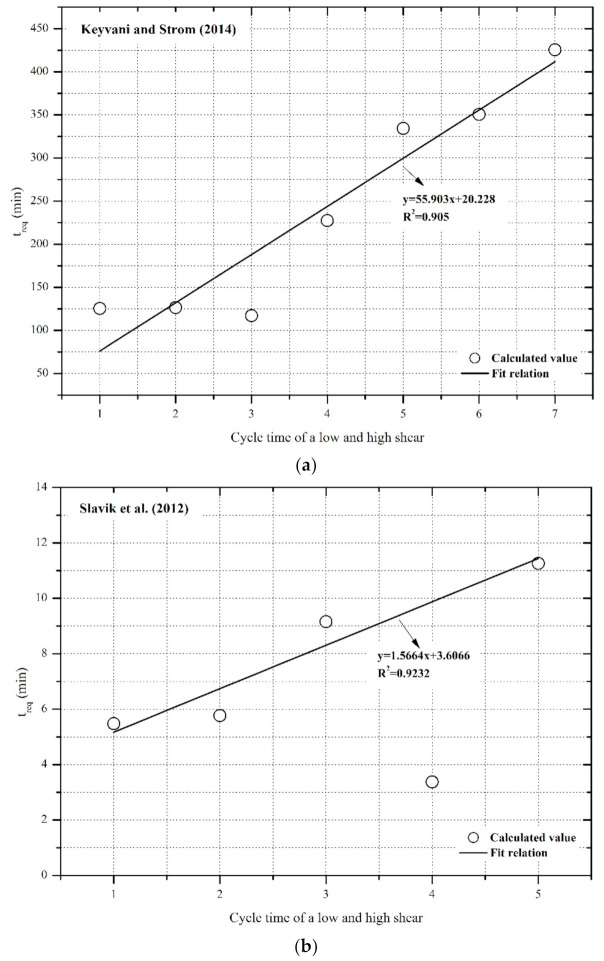
Relation between the required time $t_{req}$ and cycle times of a highly and lowly turbulent shear that the suspension was subject to in the (**a**) mud flocculation experiment of Keyvani and Strom (2014) [28]; (**b**) Fe-DOM flocculation experiment of Slavik et al. (2012) [22].

For the flocculation due to multiple cycles of a high and low turbulent shear [17,55,58,83,84], it will be interesting to see whether repeated cycles of a high and low shear play a role in affecting the floc growth pattern and the steady-state floc size from one cycle to another cycle. There are two groups of experimental results collected from the literature: one from Keyvani and Strom (2014) [28] in the cohesive sediment field and the rest from Slavik et al. (2012) [22] in the water treatment field, as shown by Figure 6 and Figure 13b in Section 3.2.1 and Section 3.2.2, respectively. In Keyvani and Strom (2014) [28], mud flocs experienced seven times cycles of a high and low turbulent shear (G = 35 s^−1^ for floc growth followed by G = 400 s^−1^ of 15 h for floc breakup), whereas Fe-DOM flocs were subject to five times cycles of a highly and lowly turbulent shear (G = 40 s^−1^ for floc growth followed by G = 1000 s^−1^ of 1 min for floc breakup) in Slavik et al. (2012) [22]. Despite different experimental conditions, both found that multiple cycles of high and low shear conditions play no obvious role in affecting the equilibrium floc size. In other words, the final steady-state floc size is only dependent on the applied shear rate, as also shown by Figure 6 and Figure 13b. However, for each floc re-growth phase of the cycle, the floc re-growth rate decreased with the number of times that the flocculation system was subject to the high shear condition. We attempted to estimate the time required for the attainment of a steady-state floc size for each floc growth phase of the cycle and to relate it to the times that the suspension was exposed to cycles of a varied shear rate. In the entropic model (Equation (1)), the required time $t_{req}$ for approaching the steady state was almost approximated by $3M/\left(L_{s}-L_{0}\right)$, as also mentioned in Section 2.1.1. Figure 23a,b shows the relation between the calculated time $t_{req}$ using the M values in Table 1 and Table 2 and cycle times of a highly and lowly turbulent shear. It can be seen from Figure 23a that there is an obvious increasing trend of the required time with cycling shear times. A similar trend is also observed in Figure 2b except for the fourth floc growth phase, which could be regarded as an outlier because of a lower floc size plateau value compared to other floc growth phases that might be due to measurement uncertainty, as shown by the sky-blue color in Figure 13b. This confirms that, as multiple cycles of high and low turbulent shear proceed, the floc re-growth rate decreases and the floc growth needs more time to approach its steady state.

Regarding the proposed entropic model due to a piecewise varied shear rate, i.e., Equation (11) or Equation (12), specifically, if the same time interval Δt is adopted, i.e., $t_{i}=i*\Delta t$ and $\left|L_{s,i}-L_{i-1}\right|/M_{i}$ is assumed to keep a constant α for each of i-th flocculation stage, floc size becomes after calculating the first-step flocculation stage based on Equation (10):

(18) $$L_{1}=\left[1-\exp\left(-\alpha \Delta t\right)\right]L_{s,1}+\exp\left(-\alpha \Delta t\right)*L_{0}$$

Then, after the second-step flocculation stage, floc size gets:

(19) $$L_{2}=\left[1-\exp\left(-\alpha \Delta t\right)\right]L_{s,2}+\exp\left(-\alpha \Delta t\right)*L_{1}$$

Substituting Equation (18) into Equation (19) can yield:

(20) $$L_{2}=\left[1-\exp\left(-\alpha \Delta t\right)\right]\left[L_{s,2}+\exp\left(-\alpha \Delta t\right)*L_{s,1}\right]+\exp\left(-2\alpha \Delta t\right)*L_{0}$$

Similarly, for the n-th flocculation period, floc size can be estimated by the following expression:

(21) $$L_{n}=\left[1-\exp\left(-\alpha \Delta t\right)\right]\sum_{i=1}^{n}\exp\left[-\left(n-i\right)\alpha \Delta t\right]*L_{s,i}+\exp\left(-n\alpha \Delta t\right)*L_{0}$$

At a large n, the second term in the right-hand side of Equation (21) approaches rapidly 0, which indicates that the initial size distribution of the floc population plays little role in determining the floc size at a later flocculation stage. As n = 1, Equation (21) can reduce to Equation (10).

### 4.4. Application of the Entropic Model in Engineering Practices and Its Limitations

In the governing equations for the three-dimensional transport of cohesive sediment, one of the most important parameters is the floc settling velocity [12,15,85,86,87,88,89,90]. It depends on the floc’s physical properties (size, shape, effective density, etc.) and the bulk properties (fluid viscosity, particle concentration, fluid turbulent condition) [4,91,92]. Flocculation models, either a Lagrangian Winterwerp model (1998) and the modified version [2,9,30,31,32], which can track the temporal evolution of a simple representative floc size, or a PBM model [35,37,93,94], which solves the aggregation and breakage processes of multi-class floc sizes by different mathematical methods, can be adopted to calculate a dynamic floc settling flux, and they are applicable to be coupled into the present mature hydrodynamic models to model the suspended sediment transport in estuarine and coastal waters [95].

The entropic model proposed in this study, which is analogous to the Winterwerp model, mainly mimics the variation of a representative floc size with flocculation time with less computational cost. Compared with the Winterwerp model and the PBM, it contains few input parameters, has a simple mathematical form, and avoids a complicated mathematical iteration; compared with the work of Zhu (2018), it can be applicable for particle flocculation kinetics’ estimation in a varied flow shear environment. This is especially true when the water–sediment system is frequently subject to multiple cycles of high and low shear rates in the river mouth, tidal estuary, and coastal waters [17,52,53,54,55,56], for which other models could require more computational works to predict the floc size distribution [83,96]. Some empirical parameters in the entropic model can be calibrated based on limited experimental or field observational data to account for the physical, chemical, and biological properties of particles and fluid.

However, it points out that some physical mechanisms regarding the aggregation and the breakage of cohesive particles, which are present in the Winterwerp model and the PBM, have not been incorporated into this entropic model, which could weaken the theoretical basis for its application. Nevertheless, it is worthy of noting that, besides the classic deterministic flocculation models, formulating a simple and accurate flocculation model to simulate the particle–particle interaction in a turbulent flow in a stochastic manner is receiving more attention from many researchers. This includes, for example, a stochastic Lagrangian flocculation model proposed by Maggi (2008), a new Monte Carlo-based flocculation model by considering the floc breakage coefficient as a random number having a log-normal distribution by Shin et al. (2015), and a quasi-Monte Carlo (QMC)-based PBM for simulating the sediment flocculation recently proposed by Shen et al. (2021). The proposed entropic model could provide a new stochastic manner to estimate the floc size variation, especially in a piecewise varied shear environment from a statistical perspective. This model could be promising, as an addition to existing flocculation models, to be coupled into present mature hydrodynamic models to model the cohesive sediment transport in estuarine and coastal regions. Adding more physical mechanism involvement into the entropic model and testing it with more experimental and/or observational results, especially in a complicated shear environment and for various flocculation processes (e.g., bio-flocculation [7,97,98,99,100], should be a focus in future research.

## 5. Concluding Remarks

In this study, an extended mathematical expression (Equation (11)) for the temporal evolution of a representative floc size (characterized by the median size of floc size distribution) of cohesive particles when the flocculation system is subject to a piecewise varied shear rate was derived by the probability methods based on the Shannon entropy theory following the work of Zhu (2018). This expression contains only the initial value and the steady-state values of floc size, as well as a parameter characterizing the maximum capacity for floc size increase (or decay) proposed in this study, and it can be adopted to track well a monotonic flow growth (or decay) pattern. Comparison with 13 flocculation experimental data from the cohesive sediment field, the wastewater treatment field, and the colloidal science field showed the validity of the proposed entropic model with a large correlation coefficient and few errors.

Furthermore, for the case of tapered shear flocculation, as presented in Spicer et al. (1998) [23], an empirically negative power function was found to fit the relation between the calculated capacity parameter and the shear rate well, which is similar to the dependence of the steady-state floc size on the shear rate. With these, the entropic model was further parameterized, and its sensitivity to all four parameters was tested: The model is more sensitive to two coefficients that have been incorporated into the capacity parameter than those in the steady-state floc size.

Compared with the classic Winterwerp model and PBM, as well as the single-step flocculation work of Zhu (2018), the entropic model contains fewer input parameters, has a simple mathematical form, and avoids large mathematical iteration works, especially when the flocculation system is frequently subject to multiple cycles of high and low shear rates as in river mouth, tidal estuary, and coastal waters. Although not many physical flocculation mechanisms were involved in this model, it makes a step toward formulating the flocculation dynamics in a stochastic and statistical manner, and it could be a potential as a good addition to existing flocculation models to be coupled into present mature hydrodynamic models to model the cohesive sediment transport in estuarine and coastal regions.

## Data Availability

Data is contained within the article.
